# Deciphering flavonoids and terpenoids biosynthesis through chromosomal-level genome, metabolome, and transcriptome integration in *Spuriopimpinella brachycarpa*

**DOI:** 10.1093/hr/uhag107

**Published:** 2026-03-30

**Authors:** Qian Zhao, Fu Wang, Yiqiao Ma, Shuyao Li, Ruidong Sun, Peng Di, Lei Gong, Xiujuan Lei, Bao Liu, Aisheng Xiong, Jian Zhang

**Affiliations:** Faculty of Agronomy, Jilin Agricultural University, Changchun 130118, China; Faculty of Agronomy, Jilin Agricultural University, Changchun 130118, China; Faculty of Agronomy, Jilin Agricultural University, Changchun 130118, China; Jilin Academy of Vegetable and Flower Science, Changchun 130033, China; Faculty of Agronomy, Jilin Agricultural University, Changchun 130118, China; Faculty of Agronomy, Jilin Agricultural University, Changchun 130118, China; College of Chinese Medicinal Materials, Jilin Agricultural University, Changchun 130118, China; Key Laboratory of Molecular Epigenetics of the Ministry of Education (MOE), Northeast Normal University, Changchun 130024, China; College of Chinese Medicinal Materials, Jilin Agricultural University, Changchun 130118, China; Key Laboratory of Molecular Epigenetics of the Ministry of Education (MOE), Northeast Normal University, Changchun 130024, China; College of Horticulture, Nanjing Agricultural University, Nanjing 210095, China; Faculty of Agronomy, Jilin Agricultural University, Changchun 130118, China; Department of Biology, University of British Columbia, Okanagan V1V1V7, Canada

## Abstract

*Spuriopimpinella brachycarpa* (2*n* = 2*x* = 22), a perennial Apiaceae herb traditionally consumed in Northeast China, is rich in bioactive compounds such as flavonoids and terpenoids and possesses both medicinal and comestible value. However, the metabolic mechanisms underlying these traits remain unclear due to the lack of genomic resources. Here, we present the first chromosome-level genome assembly of *S. brachycarpa* (4.12 Gb; scaffold N50 = 358.95 Mb; 11 chromosomes). Comparative genomics analysis revealed two postdivergence whole-genome duplication (WGD) events in Apiaceae and a close phylogenetic relationship between *S. brachycarpa* and *Daucus carota* (carrot). Metabolomic profiling indicated that flavonoids, dominated by flavanols and flavones, are most actively synthesized in leaves, with their biosynthesis likely regulated by the MYB transcription factor *SbraChr11G00348720.1*. Terpenoids, primarily monoterpenes and sesquiterpenes, accumulated predominantly under cultivated conditions, demonstrating habitat-specific patterns. Transcriptomic analysis further identified two key terpene synthase genes—*SbraChr6G00204720.1* (TPS-a subfamily) and *SbraChr3G00078100.1* (TPS-b subfamily)—associated with sesquiterpene and monoterpene biosynthesis, respectively. By integrative genomic, transcriptomic, and metabolomic data, this study systematically elucidates the biosynthesis basis of major secondary metabolites in *S. brachycarpa* and provides a valuable genetic resource for comparative genomics and molecular breeding in Apiaceae crops.

## Introduction

*Spuriopimpinella brachycarpa* (commonly known as Dayeqin or Shanqincai) is a perennial aromatic herb belonging to the Apiaceae family, primarily distributed in the mountainous regions of eastern Baishan and Tonghua City, Jilin Province, China [1]. Recognized as an important biological resource, its stems and leaves are rich in nutrients (e.g. vitamins, amino acids, dietary fiber) and contain various bioactive compounds such as flavonoids and terpenoids, qualifying it as a medicinal and edible plant [2, 3]. In traditional medicine, it has been used to treat colds, indigestion, abdominal pain, and cough [4]. Modern pharmacological studies further indicate that its extracts possess multiple bioactivities, including hepatoprotective, lipid-regulating, anti-neuroinflammatory, and antioxidant effects [5–8].

Flavonoids and terpenoids are particularly abundant in *S. brachycarpa* and contribute to its antimicrobial, antioxidant, and anti-proliferative properties [9–11]. Flavonoid biosynthesis begins with the phenylpropanoid pathway, in which phenylalanine is converted into chalcone—the central precursor for diverse flavonoid structures [12]. Terpenoids, another major class of secondary metabolites in this species, are synthesized via the mevalonate (MVA) and methylerythritol phosphate (MEP) pathways, which generate the universal five-carbon precursors isopentenyl pyrophosphate (IPP) and dimethylallyl pyrophosphate (DMAPP) [13, 14]. Terpenoid synthase (TPS) than catalyze the condensation of these precursors into various terpenoid skeletons [15]. Despite its evident value, genetic studies on *S. brachycarpa* remains preliminary, and systematic investigations into the regulatory mechanisms underlying its key bioactive compounds are still lacking.

Recent genomic and transcriptomic studies in Apiaceae crops have elucidated important agronomic traits such as male sterility and photoperiod responses [16, 17], highlighting the utility of multiomics approaches in this family. However, genetic resources are still scarce for many aromatic Apiaceae species with medicinal and edible potential, including *S. brachycarpa*. The development of long-read sequencing and telomere-to-telomere (T2T) chromosome-level assemblies now enables in-depth dissection of molecular mechanisms underlying trait formation [18]. For instance, a T2T genome of carrot revealed features of carotenoid biosynthesis [19], and a chromosome-level genome of coriander (*Coriandrum sativum*) identified key gene families associated with aroma and flavor [20]. These resources, along with specialized Apiaceae databases [21] and integrative platforms such as the Plant Genome Integration Resource (PlantGIR) [22], are accelerating systematic research in this family. Collectively, these advances indicate that high-quality genomic data will greatly promote the genetic dissection of *S. brachycarpa* and deepen our understanding of its flavonoid and terpenoid biosynthesis.

Growing consumer demand for nutritious and health-promoting wild vegetables has increased market interest in *S. brachycarpa*. However, overharvesting has severely reduced its wild populations, making cultivation essential for large-scale production. Because the biosynthesis of plant secondary metabolites is highly susceptible to environmental factors, the content and composition of flavonoids and terpenoids may differ between wild and cultivated conditions. Thus, systematically elucidating these differences is crucial for understanding how environmental factors shape quality and for optimizing cultivation practices to improve product quality.

In this study, we constructed the first chromosome-level genome of *S. brachycarpa* by integrating PacBio HiFi sequencing, Hi-C chromosome conformation capture, and next-generation sequencing. Using this genomic resource, we performed a systematic transcriptomics and metabolomics analysis of flavonoids and terpenoids in young leaves and petioles from wild and cultivated plants. This multiomics approach aimed to uncover the differential mechanisms underlying the synthesis and accumulation of these bioactive compounds under different growth conditions. Our work provides a valuable genetic resource for comparative genomics in Apiaceae, supports research on phylogenetic evolution and functional gene diversification, and establishes a molecular foundation for the genetic improvement of *S. brachycarpa* and the targeted regulation of its bioactive compounds, thereby promoting the sustainable development and utilization of this medicinal and edible plant resource.

## Results

### Chromosome-level genome assembly of *S. brachycarpa*

**Figure 1:**
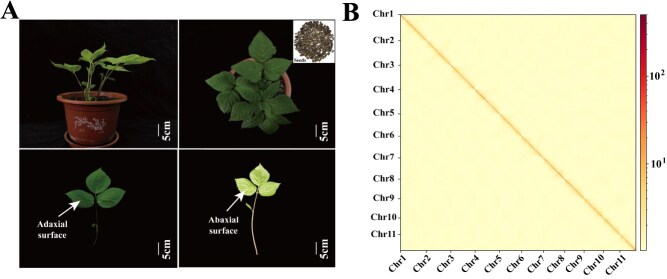
Overview of the chromosomal-level genome of *S. brachycarpa*. (A) Different organs of *S. brachycarpa*. (B) Hi-C heat map of *S. brachycarpa* chromosome interactions.

Wild *S. brachycarpa* plant were used for genome sequencing and assembly (Fig. 1A). Karyotype analysis confirmed 11 pairs of chromosomes (Fig. S1). Preliminary genome size and heterozygosity were estimated by combining next-generation sequencing data with *K*-mer analysis. At a *K*-mer size of 19, the depth-frequency distribution showed a clear bimodal pattern corresponding to the heterozygous peak (Fig. S2). Based on the total number of valid *K*-mers and the depth of the main peak, the genome size was estimated to be approximately 3480.66 Mb. Fitting a bimodal model to the *K*-mer frequency curve further yielded a heterozygosity rate of 1.89% (Table S1).

**Figure 2:**
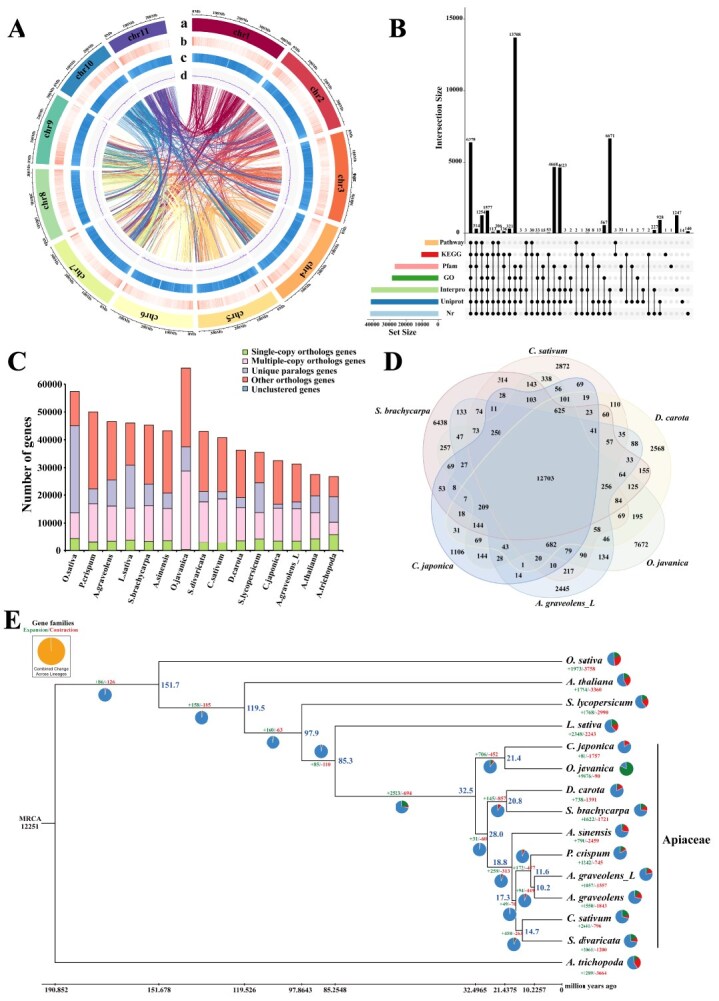
High-quality *S. brachycarpa* genome assembly and comparative genomic analysis of the *S. brachycarpa* genome. (A) Distribution of *S. brachycarpa* genomic features: (a) assembled chromosomes, (b) gene density, (c) repeats density, and (d) GC content (window size = 50 kb). Lines in the center of the circle indicate syntenic blocks. (B) Gene function annotation of *S. brachycarpa* genome. (C) Number of homologous genes shared by different species. (D) Venn diagram of gene family clustering. The numbers represent the number of gene families. Linked letters indicate the gene families shared by the indicated species, whereas single letters represent gene families specific to one species. (E) Estimation of divergence time and gene family expansion/contraction. Numbers next to each branch node represent the estimated divergence time; distinct numbers represent the expansion and contraction of gene families, respectively.

PacBio HiFi sequencing produced 7 703 466 high-quality reads, totaling 133.44 Gb with a mean read length of 17 326 bp and an N50 of 17 123 bp (Table S2). Illumina sequencing generated 205.21 Gb of raw data; after quality control, 1 355 870 208 clean reads (202.62 Gb) were retained (Table S3). A draft genome of 4.12 Gb was assembled, with a contig N50 of 25.82 Mb. Using 346.45 Gb of Hi-C data, 3.83 Gb (93.26% of the assembly) was anchored onto 11 chromosomes, comprising 399 contigs (Tables S4 and S5). The Hi-C interaction heatmap generated with HiCExplore displayed clear interaction signals between adjacent chromosomal regions, supporting the high accuracy and continuity of the assembly (Fig. 1B). After final polishing, the assembled genome size reached 4.12 Gb with a scaffold N50 of 358 95 Mb, organized into 11 chromosomes (Tables 1, S6 and Fig. 2A).

**Table 1:** Summary of chromosomal-level genome assembly of S. *brachycarpa.*

Parameters	S. *brachycarpa*
Genome size, Gb	4.12
Contig number	399
Contig N50, Mb	25.82
Scaffold number	2785
Scaffold N50, Mb	358.95
GC content, %	34.77
BUSCOs, %	95
QV	27.73

### Genome evaluation of *S. brachycarpa*

To assess the quality of the genome assembly, we performed a series of evaluations for accuracy and completeness. First, clean IIIumina reads were aligned to the assembled genome, yielding a mapping rate of 99.99% and 97.17% coverage at an average depth of 47.78× (Table S7), indicating high concordance between the sequencing data and the assembly. PacBio HiFi reads were also mapped back to the genome, achieving a mapping rate of 99.98% with 100% coverage and an average depth of 31.68×; of these, 99.4% and 95.72% of bases were covered at ≥4× and ≥10× depth, respectively (Table S8). These results further support the accuracy and continuity of the assembly at long-read resolution. Base-level consensus quality, assessed by *k-mer* analysis, gave a QV of 27.73. Gene-space completeness was evaluated with BUSCO using the eudicots_odb10 dataset (*n* = 2326), showing 95% completeness: 2209 complete orthologs (83.3% single-copy, 11.7% duplicated), while only 0.5% fragmented and 4.5% missing (Table S9). Together, these metrics demonstrate that the assembled genome is high accurate, contiguous, and effectively captures core eukaryotic gene regions.

### Genome annotation of *S. brachycarpa*

To comprehensively characterize the *S. brachycarpa* genome, we performed systematic annotation of repetitive sequences, gene structures and functions, and noncoding RNAs (ncRNAs). Multiple approaches were combined to identify repetitive sequences. After integration and removal of redundancies, a total of 3.42 Gb of transposable elements (TEs) were annotated, representing 83.35% of the genome. Long-terminal repeat (LTR) retrotransposons constituted the largest fraction of TEs (48.69%) and were further classified as LTR-Gypsy (594.87 Mb, 14.48%) and LTR-Copia (1.40 Gb, 34.04%) based on structure and transposition mechanism. A small proportion of tandem repeats (0.05%) were also detected, including satellite DNA (0.01%) and microsatellite DNA (0.04%) (Table S10, Fig. S3).

Gene structures were predicted using an integrated approach that combined *ab initio* prediction, homology-based prediction (with genomes of *Apium graveolens* (celery), carrot, coriander, and *Salvia miltiorrhiza*), and transcriptome-supported evidence. After merging redundant models, 45 250 protein-coding genes were retained. The average mRNA length was 5093.86 bp, with an average coding sequence (CDS) length of 1082.67 bp, exon length of 330.03 bp, and intron length of 1000.09 bp; gene contained an average of 4.57 exons (Table S11). Comparative analysis of gene-length distribution revealed certain differences between *S. brachycarpa* and celery or *S. miltiorrhiza*, whereas CDS, exon, and intron lengths were relatively conserved across these species (Fig. S4).

Functional annotation of the predicted genes was performed by searching for sequence and motif similarities against multiple public databases. Of the 45 250 genes, 43 345 (95.79%) obtained at least one functional annotation. Specifically, 10 436 (23.06%) genes were annotated in KEGG, 42029 (92.88%) in Nr, 41 649 (92.04%) in Uniprot, 28 596 (63.20%) in GO, 26886 (59.42%) in Pfam, and 41 471 (91.65%) in InterPro (Table S12, Fig. 2B).

ncRNAs—including miRNAs, tRNAs, rRNAs, and snRNAs—are functional RNA molecules that do not encode proteins. A total of 202 miRNAs were annotated, with an average length of 131 bp, accounting for 0.000644% of the genome. Based on structural features, 1533 tRNAs were identified, averaging 76 bp in length and representing 0.002836% of the genome. Using rRNA and Pfam databases, we further annotated 11 557 rRNAs and 8229 snRNAs, which occupied 0.106734% and 0.022813% of the genome, respectively (Table S13).

### Comparative genomic analysis

We selected 10 Apiaceae species—*S. brachycarpa*, *Petroselinum crispum*, *Anethum graveolens* (dill), *Angelica sinensis*, *Oenanthe javanica*, *Saposhnikovia divaricata*, coriander, carrot, *Cryptotaenia japonica*, and celery—along with five outgroup species (*L. sativa*, *Solanum lycopersicum*, *Arabidopsis thaliana*, *Oryza sativa*, and *Amborella trichopoda*) for ortholog identification, gene family clustering, and functional enrichment analysis (Fig. 2C). A total of 88 104 ortholog groups were identified, comprising 627 964 genes. Among these, 7532 core ortholog groups (containing 241 817 genes) were shared by all 15 species, and 71 single-copy orthologs were conserved across all lineages.

*S. brachycarpa* possessed 5929 species-specific gene families, encompassing 7716 genes. GO enrichment analysis of these families revealed significant terms including organonitrogen compound metabolic processes, ATP binding, purine ribonucleoside triphosphate binding, cytoplasm, and transition metal ion binding. KEGG pathway analysis indicated enrichment in biosynthesis of amino acids, glycolysis/gluconeogenesis, glycerolipid metabolism, RNA degradation, and ribosome biogenesis in eukaryotes (Figs S5–S6).

To further examine gene family variation among closely related species, we compared *S. brachycarpa* with five other Apiaceae species: celery, carrot, coriander, *O. javanica*, and *C. japonica*) (Fig. 2D). A total of 12 703 gene families were shared by all six species. With regard to species-specific families, *O. javanica* had the largest number (7672), slightly higher than that of *S. brachycarpa* (5929), and substantially larger than those of celery (2445), coriander (2872), carrot (2568), and *C. japonica* (1106). The similar numbers of specific families in celery, coriander, and carrot reflect their closer phylogenetic relationships.

### Phylogeny of *S. brachycarpa*

To clarify the evolutionary relationships and divergence times between *S. brachycarpa* and related species, we constructed a phylogenetic tree and estimated the divergence timeline using single-copy orthogroups from 15 species (Fig. 2E). The results indicate that the common ancestor of these species possessed approximately 12 251 gene families. A major divergence occurred around 85.3 million years ago (Mya) between Apiaceae and *L. sativa*.

**Figure 3:**
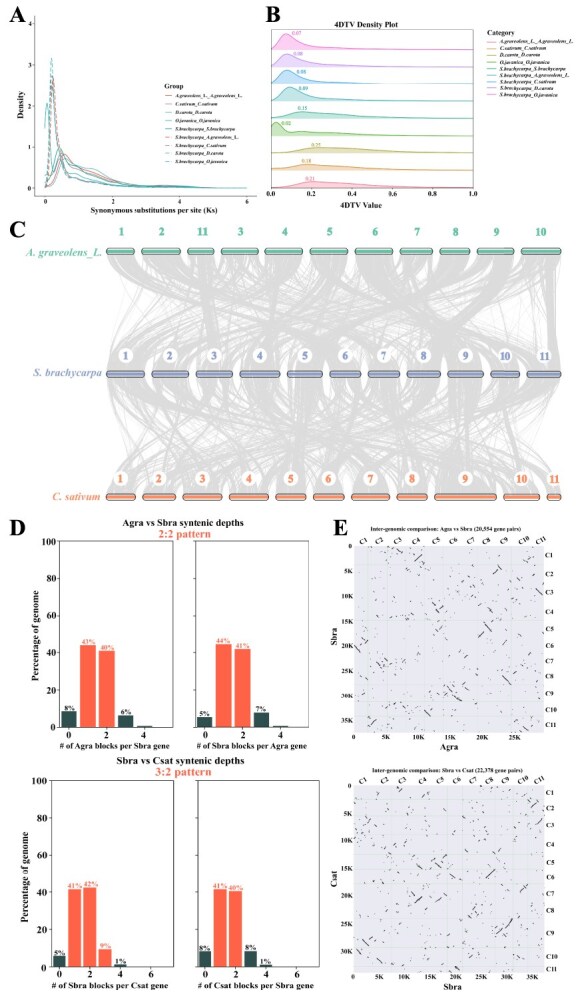
Genome evolution of the chromosomal-level genome of *S. brachycarpa*. (A) WGD analysis diagram. (B) 4DTV density diagram. (C) Collinearity diagram including *A. graveolens*_L. (Agra), *S. brachycarpa* (Sbra), and *C. sativum* (Csat). (D) Syntenic depth ratio analyses of Sbra vs Agra and Sbra vs Csat. (E) Scatter plots of Sbra vs Agra and Sbra vs Csat.

Within Apiaceae, *O. javanica* and *C. japonica* diverged early, at about 32.5 Mya, while *S. brachycarpa* diverged from other Apiaceae species—including celery and coriander—around 28.0 Mya. Among the 10 Apiaceae species, *S. brachycarpa* was most closely related to carrot, with an estimated divergence time of about 20.8 Mya, providing a temporal framework for studying the evolution of species-specific traits (Fig. 3C).

Analysis of gene family dynamics revealed 1622 expanded and 1721 contracted gene families in *S. brachycarpa*. This pattern is similar to that observed in dill (1550 expanded and 1843 contracted families), suggesting shared adaptive evolutionary trajectories. In contrast, coriander and *O. javanica* showed more pronounced gene family expansions, with 2641 and 9876 expanded families, respectively, which may reflect distinct ecological adaptations.

Functional enrichment analysis indicated that expanded gene families in *S. brachycarpa* were significantly associated with GO terms such as response to stimuli, response to stress, and defense response, highlighting their potential role in environmental adaptation. KEGG pathway analysis further revealed enrichment in plant hormone signal transduction, oxidative phosphorylation, and photosynthesis, suggesting enhanced regulation of hormone signaling, energy metabolism, and photosynthetic efficiency. Contracted gene families were enriched in carbohydrate metabolic processes, plasma membrane, and metabolic pathways (Figs S7–S10), indicating possible specialization or streamlining of genes involved in basic metabolism and cellular structure under specific evolutionary pressures.

### Whole-genome duplication in *S. brachycarpa*

To elucidate the evolutionary history of the *S. brachycarpa* genome, we investigated whole-genome duplication (WGD) events by profiling the synonymous substitution rate (Ks) distribution of homologous gene pairs (Fig. 3A). Although the Ks curve of *S. brachycarpa* showed a prominent main peak with a tail-like distribution—likely due to high genomic heterozygosity masking subtle secondary peaks—integrated analysis with four-fold synonymous third-codon transversion (4DTv) data (Fig. 3B) enable us to infer that *S. brachycarpa* has experienced two distinct WGD events: the ancestral WGD shared across Apiaceae and an additional lineage-specific WGD exclusive to *S. brachycarpa*.

To resolve the temporal dynamics of WGD events among five Apiaceae species (*S. brachycarpa*, celery, coriander, carrot, *O. javanica*), we quantified intra- and inter-species 4DTv rates (Fig. 3B). First, the intra-species 4DTv values of the five species were determined as follows: celery (0.21), coriander (0.18), carrot (0.25), *O. javanica* (0.02), and *S. brachycarpa* (0.15). Inter-species 4DTv value showed that *S. brachycarpa* diverged most recently from *O. javanica* (4DTv = 0.07), followed by carrot and coriander (0.08), and celery (0.09). Importantly, this intra-species 4DTv value (0.15) was larger than all its inter-species 4DTv values (0.07 ~ 0.09). Based on the 4DTv principle (intra-species 4DTv > inter-species 4DTv indicates WGD occurred before speciation), the peak at 0.15 represents an ancestral WGD event shared by the common ancestor of *S. brachycarpa* and its related species. Among the five species, *O. javanica* had the lowest intra-species 4DTv (0.02), corresponding to the most recent WGD, while carrot had the highest (0.25), representing the most ancient WGD. Celery and coriander displayed intermediate values (0.21 and 0.18, respectively), consistent with their phylogenetic positions in Apiaceae.

These WGD inferences were further supported by high-resolution synteny analyses (Fig. 3C–E): the 2:2 synteny depth ratio (Tables S14 and S15) between *S. brachycarpa* and celery reflects their shared ancestral WGD and conserved duplication levels across syntenic regions. In contrast, the asymmetric 3:2 synteny depth ratio (Tables S16 and S17) between *S. brachycarpa* and coriander directly indicated differential duplication patterns. Together with the 4DTv profile indicating an ancestral WGD and a recent lineage-specific WGD, this syntenic imbalance provides convergent evidence that *S. brachycarpa* underwent an additional species-specific genomic duplication during its evolution.

### Flavonoids profiles of *S. brachycarpa* across habitats

**Figure 4:**
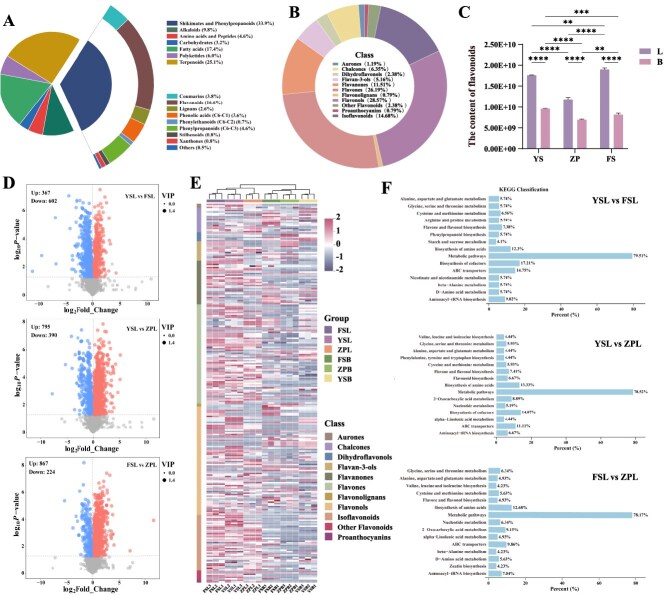
Statistical analysis of flavonoids metabolites in different tissues of *S. brachycarpa* under three habitats. (A) Classification diagram of total metabolites. (B) Circular diagram showing the metabolite composition of the flavonoids of *S. brachycarpa*. Each category is represented by a distinct sector, and the sector area indicates its proportion. (C) Flavonoid content in leaf and petiole of *S. brachycarpa* under three habitats. (D) Volcano plot in leaf of *S. brachycarpa* under three habitats. (E) Hierarchical clustering of content of flavonoids in different tissues of *S. brachycarpa* under three habitats. (F) KEGG enrichment analysis in leaf of *S. brachycarpa* under three habitats. YSL (leaf of *S. brachycarpa* under wild habitat), FSL (leaf of *S. brachycarpa* under understory bionic habitat), ZPL (leaf of *S. brachycarpa* under cultivated habitat), YSB ((Petiole of *S. brachycarpa* under wild habitat), FSB (Petiole of *S. brachycarpa* under understory bionic habitat), and ZPB (Petiole of *S. brachycarpa* under cultivated habitat).

Metabolomic analysis of leaves and petioles from wild (YS), forest-simulated (FS), and cultivated (ZP) habitats identified 1623 metabolites. Shikimates/phenylpropanoids (33.9%), terpenoids (25.1%), and fatty acids (17.4%) were the most abundant classes. Flavonoids represented the largest subgroup within phenylpropanoids (16.6%), with 11 subclasses detected; flavanones (28.57%) and flavones (26.19%) predominated (Fig. 4A and B). PCA clearly separated the six sample groups, with replicates clustering closely (Fig. S11).

Total flavonoid content ranked FS > YS > ZP and was significantly higher in leaves than in petioles (Fig. 4C). Analysis of differentially accumulated metabolites (DAMs) showed that, relative to ZP, up-regulated metabolites in YS and FS leaves outnumbered down-regulated metabolites by ~2-fold and ~4-fold, respectively, whereas more metabolites were down-regulated in the YS vs FS comparison (Fig. 4D). Clustering of DAMs confirmed that flavonoids were consistently up-regulated in leaves, with greater abundance in YS and FS leaves than in ZP leaves (Fig. 4E).

KEGG enrichment of DAMs highlighted phenylpropanoid biosynthesis, flavone/flavonol biosynthesis, and flavonoid biosynthesis pathways. In YSL vs ZPL, flavonoid biosynthesis terms accounted for 14.08% of enriched pathways and involved more genes than in other comparisons, underscoring the central role of flavonoid metabolism in habitat-specific responses (Fig. 4F).

### Flavonoid biosynthesis pathway in *S. brachycarpa*

**Figure 5:**
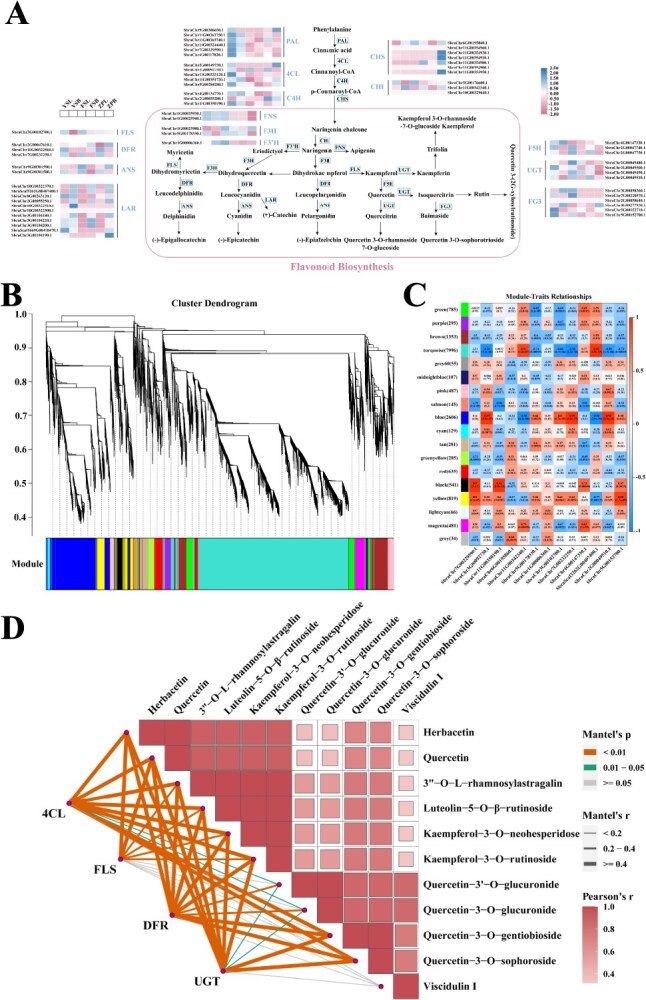
Integrated analysis of the flavonoid synthesis pathway and its regulatory network in *S. brachycarpa*. (A) Schematic diagram of the flavonoid biosynthetic pathway in *S. brachycarpa*. This figure outlines the core enzymatic steps from the general phenylpropanoid pathway to flavonoids. Key structural genes identified in the *S. brachycarpa* genome and their corresponding catalytic steps are annotated. The gradient in the adjacent bar plot represents the expression levels of the genes across the three habitats. (B) Module identification from WGCNA. The dendrogram displays the clustering of all expressed genes based on topological overlap, with the horizontal bar below indicating the module assignment for each gene. (C) Association between gene expression profiles and modules. The heatmap shows the correlation coefficients between the expression patterns of key flavonoid structural genes and the module eigengenes of each WGCNA module. One module exhibits the strongest positive correlation with most flavonoid biosynthetic genes. (D) Correlation network between co-expressed structural genes and flavonoid metabolites.

We identified 59 structural genes involved in flavonoid biosynthesis in the *S. brachycarpa* genome (Table S18). Integrated transcriptomic analysis across three habitats and two tissue types revealed distinct tissue-specific and habitat-dependent expression profiles for these genes (Fig. 5A). Within the general phenylpropanoid pathway, most *SbPAL*, *Sb4CL*, and *SbC4H* genes displayed coordinate up-regulation in the YS habitat, with six *SbPAL* genes exhibiting particularly strong leaf-preferential expression under YS conditions, suggesting habitat-modulated precursors synthesis.

Among 45 flavonoid-specific genes, five—*SbraChr11G00354960.1* (*SbCHS*), *SbraChr11G00354900.1* (*SbCHS*), *SbraChr10G00329640.1* (*SbCHI*), SbraChr10G00322500.1 (*SbLAR*), and *SbraChr2G00058640.1* (*SbFG3*)—showed no detectable expression (FPKM = 0) across all samples, indicating potential inactivity or specialized roles under the tested conditions. Chalcone synthase (CHS), a key rate-limiting enzyme, was represented by seven *SbCHS* genes, five of which were significantly up-regulated in ZP petioles. In contrast, *SbFLS* (*SbraChr3G00102700.1*) was expressed more highly in leaves, identifying leaves as the primary site for flavonol biosynthesis. Most *SbLAR* genes showed petiole-enriched expression, especially in wild condition, reflecting tissue-divergent accumulation strategies.

Other pathway genes (including *SbDFR*, *SbANS*, *SbFNS*, *SbF3H*, *SbF3’H*, *SbF5H*, and *SbFG3* exhibited pronounced expression variation across habitats and tissues, demonstrating that flavonoid biosynthesis is finely regulated by both environmental and developmental factors through heterogeneous regulatory mechanisms.

### Identification of flavonoid-related transcription factors in *S. brachycarpa*

To elucidate the regulatory mechanisms of flavonoid metabolism, we conducted weighted gene co-expression network analysis (WGCNA) on RNA-seq data from leaves and petioles across three habitats. Expressed genes were grouped into 18 co-expression modules (Fig. 5B). Network topology and sample clustering were validated prior to module detection (Figs S12 and S13), and soft-threshold selection was documented (Table S19). The blue module exhibited the strongest association with flavonoid biosynthetic genes, including *Sb4CL* (*SbraChr3G00092730.1*), *SbFLS* (*SbraChr3G00102700.1*), *SbDFR* (*SbraChr7G00232250.1*), and *SbUGT* (*SbraChr2G00049510.1*) (Fig. 5C). KEGG enrichment analysis confirmed that genes in this module were significantly enriched in flavone/flavonol and flavonoid biosynthesis pathways (Fig. S14). These genes were also up-regulated in levels compared to petioles (Fig. S15), consistent with higher flavonoid accumulation in leaves.

Correlation analysis between four core structural genes from the blue module and 11 commonly detected flavonoids revealed that *Sb4CL*, *SbDFR*, and *SbUGT* positively correlated with eight flavonoids, whereas *SbFLS* correlated only with herbacetin and quercetin (Table S20, Fig. 5D). This supports flavanols and flavones as the predominant flavonoid subclasses and suggests subclass-specific roles for these genes.

Within the blue module, we identified 48 transcription factors (TFs) from 33 families, including AP2, bHLH, C2H2, MYB, and WRKY (Table S21). Co-regulation network analysis indicated putative interactions between TFs and structural genes (Table S22, Fig. S16). Notably, *SbMYB* (*SbraChr11G00348720.1*) showed co-expression links with key flavonoid biosynthetic genes, such as *SbraChr7G00232250.1* (*DFR*), *SbraChr11G0035011090.1* (*C4H*), and *SbraChr3G00102700.1* (*FLS*), and their promoters contained conserved MYB-binding sites (Fig. S17), suggesting *SbMYB* as a candidate regulator of flavonoid biosynthesis. These results provide a foundation for further dissection of the flavonoid regulatory network in *S. brachycarpa*.

### Terpenoid biosynthesis in *S. brachycarpa*

**Figure 6:**
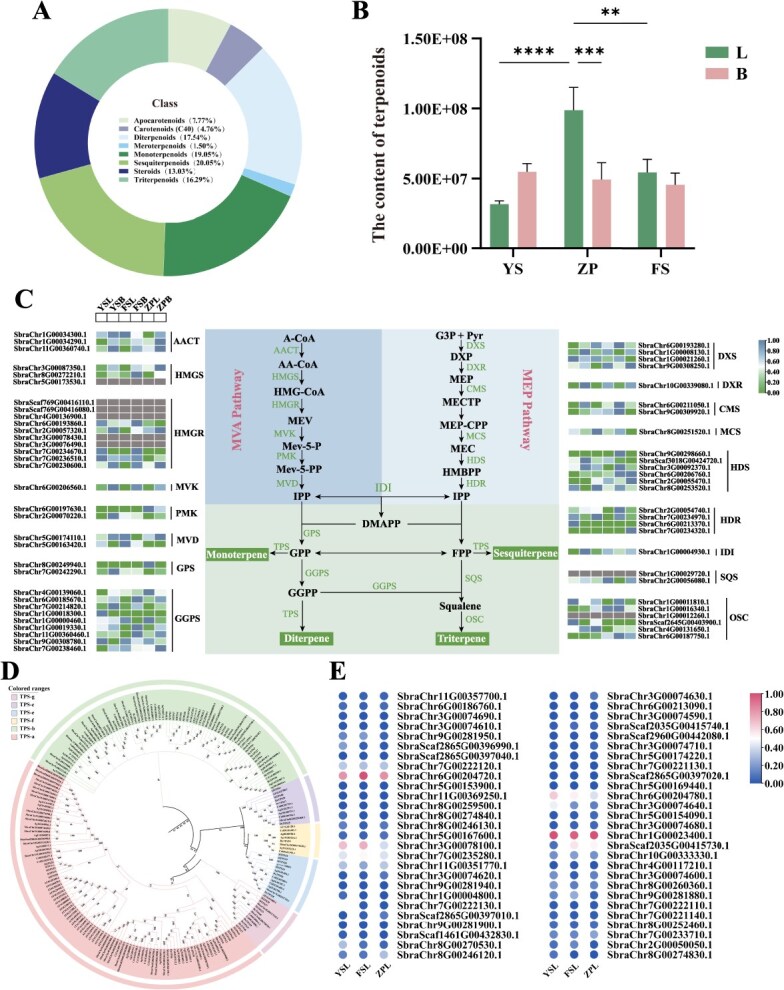
Analysis of terpenoid synthesis in *S. brachycarpa*. (A) Composition of terpenoids identified in *S. brachycarpa* tissues. The circular diagram illustrates the relative abundance of major terpenoid classes detected via untargeted metabolomics in leaf and petiole samples pooled across three habitats. Values represent the percentage of total identified terpenoids. (B) Tissue- and habitat-specific accumulation of total terpenoids. (C) Schematic overview of the terpenoid biosynthetic pathway in *S. brachycarpa*. Key enzymes and intermediates in the MEP and MVA pathways are shown. (D) Phylogenetic analysis of TPS gene family members. An unrooted maximum-likelihood tree was constructed using full-length protein sequences of TPS genes from *S. brachycarpa*, *A. graveolens* [23], *D. carota* [24], *A. thaliana* [25], *C. sativum*, and *S. lycopersicum* [26]. Major subfamilies (TPS-a to TPS-g) are indicated by distinct arcs. (E) Expression profiles of the *SbTPS* genes in leaf across three habitats. The heatmap displays FPKM values (log2-transformed) of 54 *SbTPS* genes in leaves from YS, FS, and ZP habitats.

Untargeted metabolomics identified eight terpenoid classes in *S. brachycarpa* leaves and petioles from three habitats. Sesquiterpenoids (20.05%), monoterpenoids (19.05%), diterpenoids (17.54%), and triterpenoids (16.29%) were the major constituents (Fig. 6A). Total terpenoid content showed habitat-dependent tissue variation: higher in YS petioles than leaves, but the opposite in FS and ZP. ZP leaves contained the highest overall terpenoid levels (Fig. 6B). Fourteen high-abundance terpenoids—including andrographolide (diterpenoids) and ethyl chrysanthemate (monoterpenoid), were identified, many associated with anti-inflammatory, insect-repellent, and aromatic activities (Table S23).

From the genome we identified 18 gene families involved in terpenoid synthesis. In the upstream MVA pathway, *SbHMGS* (*SbraChr5G00173530.1*) and five *SbHMGR* genes showed no detectable expression. By contrast, multiple genes in the MEP pathway (*DXS*, *DXR*, *CMS*, *MCS*, *HDS*, *HDR*) were differentially expressed across samples. Notably, one *IDI* gene was significantly higher in petioles than leaves, with peak expression in YS petioles, correlating with tissue-specific terpenoid accumulation (Fig. 6C).

Downstream, *GPS*, *GGPS*, and *TPS* family members were expressed in at least one habitat-tissue combination, contributing to monoterpenoid, sesquiterpenoid, and diterpenoid formation. For triterpenoids, two *SbSQS* and six *SbOSC* genes were identified. Several *SbOSC* genes exhibited leaf-specific up-regulation in particular habitats (YS, FS, or ZP), and two (*SbraChr2G00056080.1* and *SbraChr1G0001634*0.1) were specifically up-regulated in ZP petioles, suggesting localized roles in terpenoid synthesis.

### Identification of members of terpene synthase gene family

Fifty-four terpene synthase (TPS) genes were identified in the *S. brachycarpa* genome and classified into six subfamilies. TPS-a (29 members) and TPS-b (20 members) together accounting for 90.74% (49/54) of the family, indicating their dominance. Compared with other Apiaceae species (coriander, celery, carrot) and eudicots (*A. thaliana* and *S. lycopersicum*), *S. brachycarpa* possesses an expanded TPS repertoire (54 members), which may reflect adaptation to the local Changbai Mountain climate (Fig. 6D).

Expression analysis revealed distinct tissue- and habitat-specific patterns. In leaves, *SbraChr6G00204720.1* and *SbraChr1G00023400.*1 were up-regulated across all habitats, whereas *SbraChr3G00078100.1* and *SbraChr6G00204780.1* showed higher expression in YS and FS than in ZP. (Fig. 6E). In petioles, *SbraChr3G00078100.1* was most highly expression overall, while *SbraChr1G00023400.1* and *SbraChr6G00204720.1* exhibited habitat-specific high expression in YS and FS, respectively (Fig. S18). Expression levels of moderately and highly expressed *SbTPS* genes correlated with terpenoid contents (Fig. S19).

qRT-PCR of 16 representative *SbTPS* genes confirmed subfamily-specific tissue preferences: most TPS-a genes were leaf-enriched, whereas TPS-b, TPS-c, and TPS-g genes were predominantly expressed in petioles (Fig. S20). These results support tissue-specific roles for TPS-a and TPS-b subfamilies in leaf and petiole terpenoid synthesis. In particular, *SbraChr6G00204720.1* (TPS-a) and *SbraChr3G00078100.1* (TPS-b) are proposed as core candidates for sesquiterpenoid and monoterpenoid biosynthesis, reflecting a complementary, tissue-partitioned metabolic strategy in *S. brachycarpa*.

## Discussion

The Apiaceae family, comprising approximately 434 genera and 3700 species, holds significant economic and ecological value. Genomic resources for key members—including carrot [19, 27], celery [28], *O. javanica* [29], coriander [20], and *A. sinensis* [30]—have enabled deeper molecular insights [31–33]. To facilitate the study of *S. brachycarpa*, we generated its first high-quality, chromosome-level genome (4.12 Gb). Comparative phylogenomic analysis with ten other Apiaceae genomes estimated the Apiaceae–Asteraceae divergence at ~85.3 Mya. This estimate, primarily anchored by recent asterid calibration points, is robust to variations in deeper calibration choices and supports a mid-Cretaceous diversification concurrent with major angiosperm radiation. Furthermore, we identified two WGD events in *S. brachycarpa*: an ancestral WGD shared across Apiaceae [32, 34] and a more recent, lineage-specific WGD. These duplications likely supplied the genetic substrate for the evolution of secondary metabolism and may have facilitated the adaptation of *S. brachycarpa* to its native cold and humid habitats.

Flavonoids, particularly flavonols (e.g. kaempferol) and flavones (e.g. luteolin), were major bioactive constituents in *S. brachycarpa.* Their biosynthesis exhibited distinct tissue specificity, with leaves being the primary site for flavonol production, as evidenced by the consistent, leaf-preferential expression of *SbFLS* across habitats [35–38]. For flavone formation, plants possess two flavone synthases (FNS) types: widespread FNS II and Apiaceae-conserved FNS I, which introduces a C2–C3 double bond into flavano7nes [39, 40]. The first FNS I characterized in parsley (*PcFNS I*) [41], with homologs subsequently identified in celery [42], carrot [43], *A. thaliana*, and maize [44]. In *S. brachycarpa*, two *SbFNSI* genes displayed coordinated expression, highest in YS and FS leaves and lowest in ZP petioles, indicating regulation by both developmental and environmental cues. This pattern aligns with the role of FNS I in substrate channeling, as demonstrated in purple celery where *AgFNSI* directs naringenin towards apigenin synthesis [45], underscoring the conserved yet adaptable function of FNS I in Apiaceae flavonoid metabolism.

MYB TFs are central regulators of flavonoid biosynthesis [46]. Our multiomics analysis identified a candidate MYB-mediated module underpinning tissue-specific accumulation in *S. brachycarpa*. WGCNA co-expression analysis clustered a key *SbMYB* (*SbraChr11G00348720.1*) within the flavonoid-associated “blue” module, closely associated with biosynthetic genes, particularly *SbFLS*. Transcriptomic and metabolomic data consistently showed higher *SbFLS* expression and flavonol abundance in leaves across habitats, with a significant positive correlation between them. These results suggest that *SbMYB* promotes leaf-preferential flavonol biosynthesis via *SbFLS* activation, potentially aiding stress tolerance in aerial tissues. Although the MYB-FLS axis appears conserved in species such as tartary buckwheat and pear [47, 48], regulatory divergence exists within Apiaceae: in celery, *AgMYB12* activates *AgFNS* instead of *AgFLS*, favoring flavone (e.g. apigenin) accumulation [49, 50]. This differential targeting likely explains the distinct flavonoid profiles—flavonol-rich in *S. brachycarpa* versus flavone-rich in celery—each aligning with respective ecological or culinary adaptations.

Terpenoids play vital roles in plant defense and adaptation and hold industrial value [51]. Their structural diversity is largely shaped by TPS, which convert GPP and FPP into various skeletons [52]. In *S. brachycarpa*, we identified 54 *TPS* genes, mostly from the TPS-a (29) and TPS-b (10) subfamilies [53]. Two genes, *SbraChr6G00204720.1* (TPS-a) and *SbraChr3G00078100.1* (TPS-b), were prioritized as core candidates based on (i) tissue-specific high expression (leaf vs petiole), (ii) positive correlation with sesquiterpenoid and monoterpenoid contents, and (iii) concordance between subfamily function and product types [54]. TPS subfamily composition varies among Apiaceae species, influencing terpenoid profiles: TPS-b-rich dill and *O. javanica*, accumulate more monoterpenoids [32, 33], whereas TPS-a dominance in *S. brachycarpa* correlates with abundant sesquiterpenoids.

Notably, terpenoid content was highest in the open, cultivated (ZP) habitat. Physiologically, stronger sunlight in ZP likely enhances photosynthetic precursor supply and up-regulates TPS genes (especially TPS-a), promoting synthesis [55]. Ecologically, the exposed ZP environment subject plants to greater abiotic stress (e.g. high radiation, temperature fluctuation). As effective antioxidants, elevated terpenoids may serve an adaptive defense role by scavenging reactive oxygen species [56, 57]. This habitat-driven accumulation mechanism offers a theoretical basis for optimizing cultivation (e.g. light regulation) to enhance terpenoid yields.

## Materials and methods

### Plant materials and genome sequencing

*S. brachycarpa* plant were collected from three adjacent habitats (≤5 km apart) in the Changbai Mountain area, Jilin Province: a natural forest slope (YS), an artificially forest-simulated planting area (FS), and an open cultivated field (ZP). The YS habitat featured understory scattered light and natural dark brown soil with no human intervention. The FS habitat was designed to mimic YS conditions, sharing the same canopy shading and soil type, but with no fertilization or irrigation. The ZP habitat was fully sun-exposed, tilled to local dark brown soil, and received only regular irrigation to maintain moisture.

High-quality genomic DNA was extracted from young leaves of wild *S. brachycarpa* using an optimized CTAB method [58]. After quality assessment, the DNA was sheared for library construction. An Illumina paired-end library was prepared with the Nextera DNA Flex Library Prep Kit (Illumina, San Diego, CA, USA) and sequenced on the BGISEQ-500 platform [59]. For long-read sequencing, a PCR-free SMRTbell library was constructed and sequenced on the PacBio Sequel II system [60]. Hi-C libraries were prepared by cross-linking chromatin with formaldehyde, followed by restriction digestion, biotin labeling, proximity ligation, and streptavidin-based capture of spatially interacting fragments; after quality control, these were sequenced on an Illumina platform [61]. All sequencing services were provided by Wuhan Benagen Tech Co., Ltd (Wuhan, China).

### Karyotype analysis

Karyotype analysis was conducted following a published protocol [62] with slight modifications. Wild *S. brachycarpa* plants were collected with native soil and acclimatized in a greenhouse for about 20 days until new roots appeared. Root tips (0.5 cm) were excised and placed in a perforated 0.5-ml microcentrifuge tube. Chromosomes were condensed by exposing the tubes to nitrous oxide gas for 2 h, followed by fixation in 90% glacial acetic acid for 5 min. After removal of the acetic acid, samples were rinsed with ddH₂O and transferred into a mixed enzyme solution containing cellulase and pectinase (2:1 ratio) for cell wall digestion at 37°C for 30 min. The reaction was stopped by washing with 75% ethanol. After discarding the supernatant, about 30–40 μl of ethanol was retained, and the tissue was gently ground to release cells. The suspension was vortexed briefly and centrifuged at 7000 rpm for 90 s at 25°C. The pellet was air-dried on ice, resuspended in 20–30 μl of glacial acetic acid, and 10 μl of the suspension was dropped onto a moistened glass slide. After air-drying at room temperature for 5–10 min, chromosomes were examined and imaged under an optical microscope equipped with a digital imaging system for karyotype analysis.

### Genome assembly

PacBio SMRT sequencing raw data were processed with SMRTLink 8.0 [63] (parameters: −min-passes = 3; −min-rq = 0.99) to generate high-fidelity (HiFi) reads. These reads were assembled into contigs using Hifiasm (v0.14.2) [64]. Hi-C raw data were processed and normalized with HICUP (v0.8.0) [65] to obtain valid chromatin interaction pairs. These interaction data were then used in Juicebox (v1.11.08) [66] to order, orient, and scaffold contigs into chromosome-level sequences by visualizing the Hi-C interaction heatmap.

### Genomic evaluation

Raw NGS data base-called and converted to FASTQ format. Quality control was performed with FastQC (v0.11.9) [67]. Genome size and heterozygosity were estimated by analyzing the *K*-mer frequency distribution using the kmer_freq subroutine in GCE (v1.0.0) [68], which incorporates the Lander–Waterman algorithm. Assembly continuity was assessed using contig N50. Accuracy was evaluated by mapping clean reads to the assembly with BWA [69] and calculating mapping rate and coverage. Completeness was assessed with BUSCO (v5.beta.1) [70] using the parameter: “-evalue 1e^-5^.”

### Genome annotation

Repetitive sequences were annotated by homology-based searching against RepBase using RepeatMasker (vopen-4.0.9) [71] and by *de novo* prediction with RepeatModeler (vopen-1.0.1) [72]; results were merged and deduplicated. Gene structures were predicted by integrating homology-based evidence (using protein from celery, carrot, coriander, and *S. miltiorrhiza*), *ab initio* prediction, and transcriptomic support. Functional annotation was performed by searching for sequence and motif similarities against KEGG [73], Nr [74], UniProt [75], GO [76], Pfam [77], and InterPro [78]. Noncoding RNAs were identified using: tRNAscan-SE (v1.23) for tRNA [79]; BLAST against rRNA databases for rRNA [80]; and INFERNAL (v1.1.2) with the Rfam database for snRNA, miRNA, and other ncRNAs [81, 82]. Annotation completeness was assessed using BUSCO (v5.2.2) [83].

### Gene family identification, expansion, and contraction

Protein sequences from *S. brachycarpa* and 14 other representative species (celery, carrot, coriander, *O. javanica*, *C. japonica*, *Oenanthe sinensis*, dill, parsley (*P. crispum*), *S. divaricata*, *A. sinensis*, *L. sativa*, *A. thaliana*, *O. sativa*, and *Solanum tuberosum*) were clustered into gene families using OrthoFinder (option “-M msa”) [84]. Single-copy and multicopy families were identified from BLASTp alignments (v2.6.0; parameters: −evalue 1e-5; −outfmt 6) [85], which applies a birth–death model to infer ancestral family sizes and detect significant expansion/contractions across lineages [86]. Functional enrichment of species-specific and shared families was conducted using clusterProfiler [87].

### Phylogenetic analysis and dating

A phylogenetic tree was reconstructed from aligned single-copy protein sequences (MUSCLE v3.8.31) [88] after filtering poorly aligned regions (trimal v1.2rev59; parameter: -gt 0.2) [89]. A maximum-likelihood tree was built with RAxML (v8.2.10) [90]. Divergence times were estimated using mcmctree in PAML (v4.9), with calibration applied to five nodes (uniform prior) using TimeTree-derived bounds: *S. polyrhiza*–*O. sativa* (142.1–163.5 Mya), *S. polyrhiza*–*A. trichopoda* (179.9–205.0 Mya), *S. polyrhiza*–*A. thaliana* (111.4–123.9 Mya), dill–*L. sativa* (76.0–97.4 Mya), and dill–carrot (20.1–40.6 Mya). Parameters were nsample = 3 000 000, burnin = 8 000 000, seqtype = 0, and model = 4. WGD analysis involved identifying intra-genomic collinear blocks with MCScanX (v0.8) [91], calculating synonymous (Ks) and nonsynonymous (Ka) substitution rates (yn00 in PAML (v4.9)), and plotting Ks distributions (ggplot2 v2.2.1). 4DTv values were computed with ParaAT (v2.0) [92] to validate WGD signals. Positive selection was assessed using CodeML in PAML (v4.9) [93].

### Metabolomics sequencing and analysis

Young leaf (third-fourth fully expanded) and petiole (~2.5 cm) tissues of *S. brachycarpa* were collected in mid-June (9:00–10:00 a.m.) from three habitats (YS, FS, and ZP) in the Changbai Mountain. Plants were standardized (height 12 ± 2 cm, stem diameter 2 ± 0.5 mm) and three biological replicates were taken per type. Tissues were frozen in liquid nitrogen, freeze-dried, and ground into a fine powder. For extraction, 25 mg powder was mixed with 1000 μl of cold extraction solution (methanol:acetonitrile:water = 2:2:1, v/v/v) containing internal standards, homogenized (35 Hz, 4 min), ultrasonicated on ice (5 min, three cycles), incubated at −40°C for 1 h, and centrifuged (12 000 rpm, 15 min, 4°C). The supernatant was filtered (0.22 μm) for analysis. Metabolites were separated on a Phenomenex Kinetex C18 column (2.1 × 50 mm, 2.6 μm; 40°C) using a Vanquish UHPLC system with a water-acetic acid (0.01%) and isopropanol-acetonitrile (1:1, v/v) gradient. Detection was performed on an Orbitrap Exploris 120 mass spectrometer in positive/negative ionization modes (full-scan and MS/MS). Quality control samples were analyzed every 10 injections.

### Transcriptome sequencing and analysis

Total RNA was extracted using the TransZol Up Plus RNA Kit (TransGen Biotech, Beijing, China) and assessed for integrity, concentration and purity. High-quality RNA was used to construct cDNA libraries (TruSeq RNA Library Prep Kit, Illumina), which were sequenced on the Illumina NovaSeq 6000 platform. Raw reads were processed with fastp (v0.21.0; parameter: -l 30) to remove adapter and low-quality bases, followed by quality assessment with FastQC (v0.11.9) [94]. Clean reads were aligned to the reference genome using STAR (v2.7.9a) [95], transcripts were generated with StringTie (v2.1.4) [96], and expression levels (FPKM) were quantified with RSEM (v1.3.1) [97]. Differential expression analysis was performed with DESeq2 (v1.34.0) [98], with significantly differentially expressed genes defined as those with padj <0.05 and |log₂fold change| ≥ 1.

### Identification of structural genes in flavonoid and terpenoid biosynthesis

Based on LC–MS/MS metabolomic data, flavonoids, and terpenoids were identified by matching to standard databases and MS/MS interpretation. Using genome annotation data and BLAST comparisons (e-value <10^−5^) against *A. thaliana* reference proteins, we systematically identified 15 flavonoid-related gene families (PAL, 4CL, C4H, CHS, CHI, F3H, F3’H, FNS, FLS, DFR, ANS, LAR, F5H, UGT, and FG3). Terpenoid-related gene were similarly identified, covering the MVA pathway (AACT, HMGS, HMGR, MVK, PMK, and MVD), the MEP pathway (DXS, DXR, CMS, MCS, HDS, HDR), and skeleton-forming genes (IDI, GPS, GGPS, SQS, OSC). All candidates were validated for conserved domains using Pfam database. Transcriptomic profiles of these genes in leaves and petioles across habitats were analyzed to elucidate environment-mediated regulation of secondary metabolites.

### Transcription factor prediction and co-expression network construction

Using FPKM expression data of flavonoid biosynthetic genes, low- or stably-expressed genes were filtered out (genefilter R package, threshold = 0.8). A WGCNA was built with soft-threshold power β = 14, minimum module size = 50, and merging threshold = 0.25. Transcription factors were annotated using PlantTFDB database [99]. protein–protein interactions networks were predicted with the STRING database (http://string-db.org) [100], and visualized in Cytoscape (v3.10.3) [101]. Differentially accumulated flavonoids (FDR < 0.05, |log2FoldChange| ≥ 1) common across habitats were selected, and their correlations with structural genes were evaluated using the Mantel test. Analyses and visualizations were performed in the R environment.

### Identification of TPS gene family members

Fifty-four *SbTPS* genes were identified from the *S. brachycarpa* genome based on characteristic TPS domains (PF01397 and PF03036). A phylogenetic tree was constructed using MEGA11 with TPS protein sequences from celery, carrot, coriander, *S. brachycarpa*, *A. thaliana*, and *S. lycopersicum* [102]. Expression patterns across habitats and tissues were analyzed with TBtools [103] and visualized in heatmaps. qRT-PCR validation was performed on leaf and petiole samples using gene-specific primers (Table S24) and ChamQ SYBR qPCR Master Mix (Vazyme). *SbActin* served as the internal control, and expression was calculated via the 2^-ΔΔCT^ method with three biological replicates [104].

## Conclusion

This study presents the first chromosome-level genome of *S. brachycarpa* and integrates comparative genomics, transcriptomics, and metabolomics to elucidate the biosynthesis of flavonoids and terpenoids. Ancient and lineage-specific duplications contributed to the diversification of secondary metabolism and environmental adaptation. The identified key genes and regulatory modules, particularly in MYB-mediated flavonoid and TPS-driven terpenoid pathways, provide valuable targets for metabolic engineering and molecular breeding in Apiaceae crops.

## Supplementary Material

Web_Material_uhag107

## Data Availability

The genome sequence data generated in this study are publicly available at the National Genomics Data Center (NGDC) (https://ngdc.cncb.ac.cn/) under BioProject accession number PRJCA056023. The metabolomic data have been deposited in the same repository under accession number OMIX014457. Raw RNA-seq data can be accessed through the NCBI database (https://www.ncbi.nlm.nih.gov/) under BioProject accession number PRJNA1402050.
