# Supplementary material for: Deciphering flavonoids and terpenoids biosynthesis through chromosomal-level genome, metabolome, and transcriptome integration in Spuriopimpinella brachycarpa
Source: Hortic Res. 2026 Mar 30;13(7):uhag107. doi: 10.1093/hr/uhag107 (PMC13291319; doi:10.1093/hr/uhag107)
Supplement: Web_Material_uhag107 [file web_material_uhag107.zip › 20260205-Supplementary Figures.docx]

**Supplementary Figures**


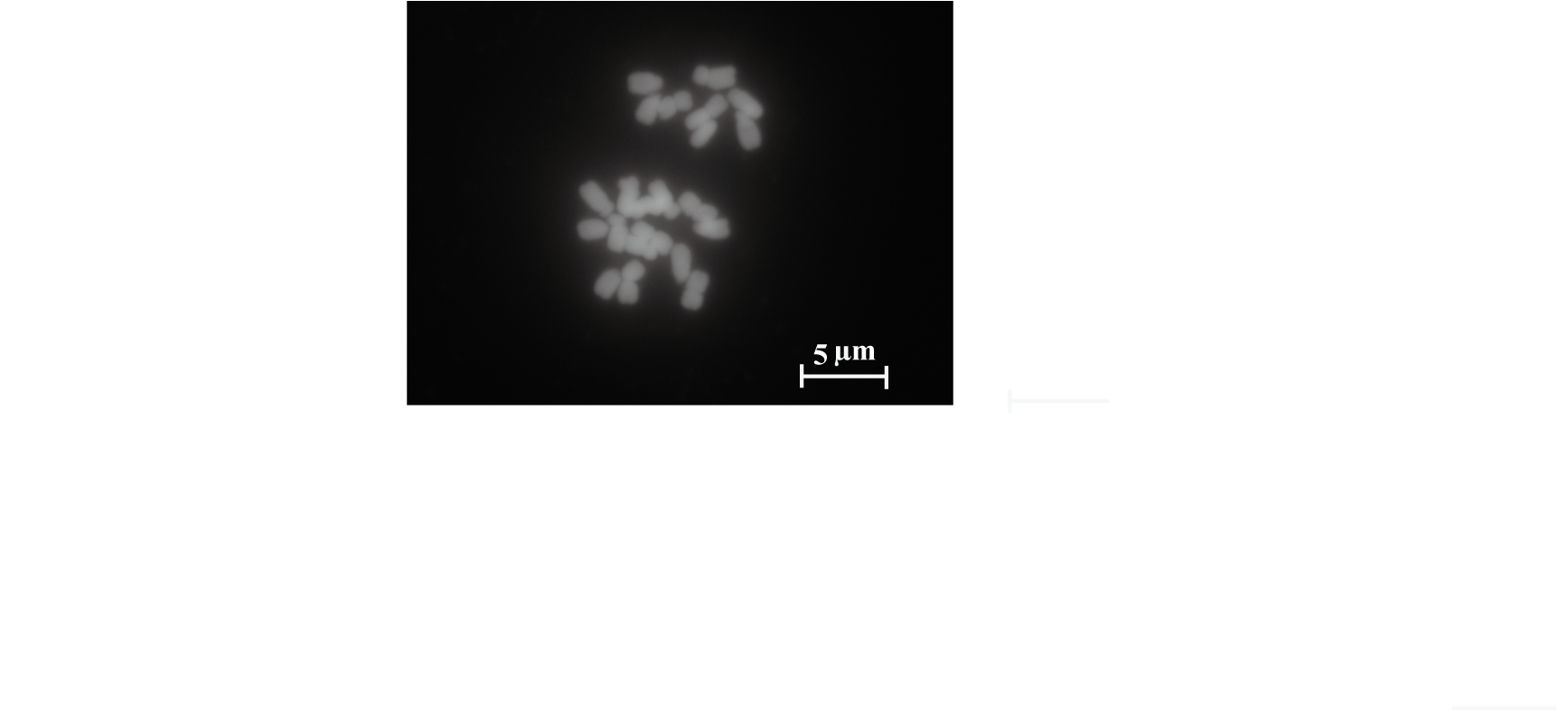


Figure S1. Karyotype analysis of *S. brachycarpa.*


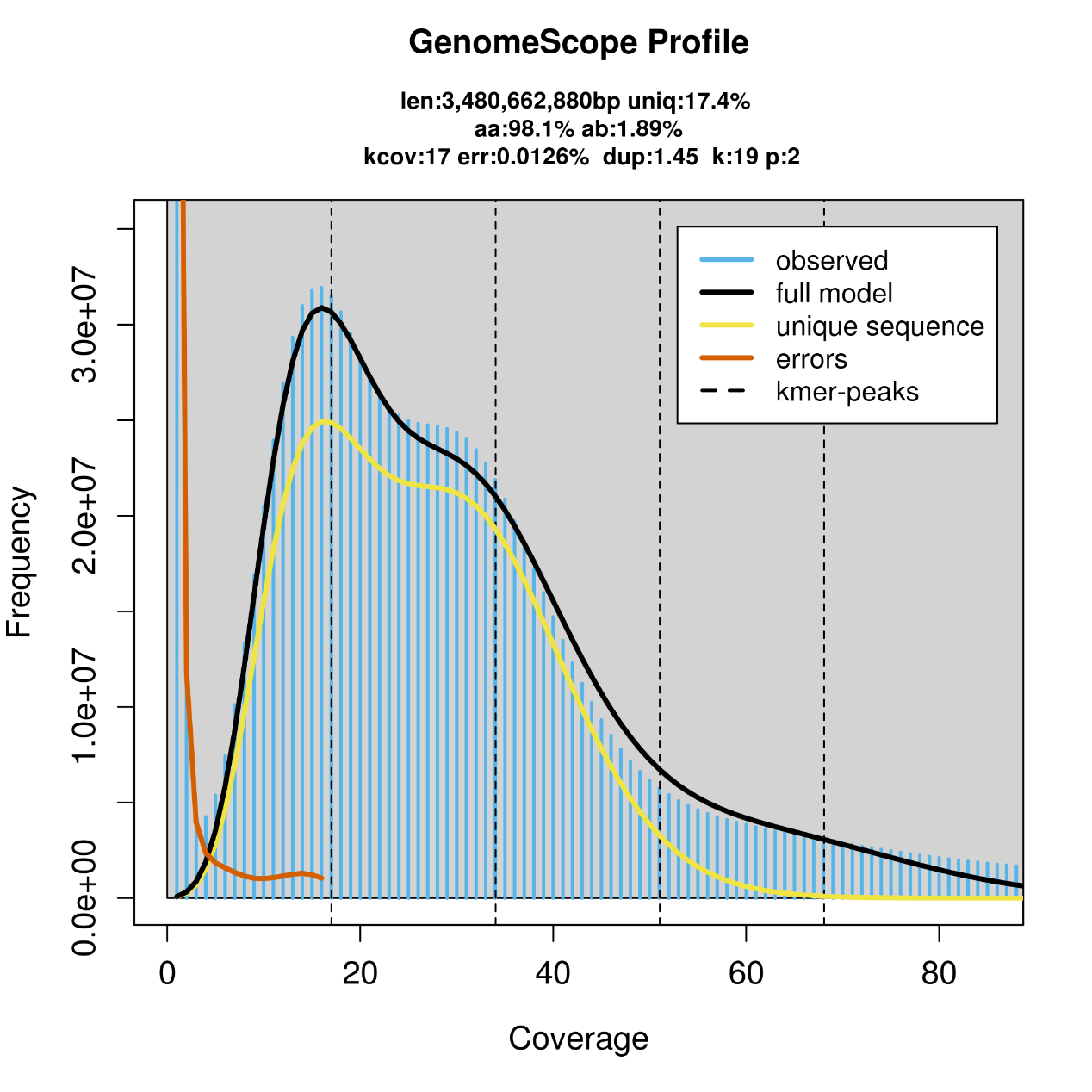


Figure S2. *K-*mer depth-frequency distribution *(K-*mer = 19*).*

**
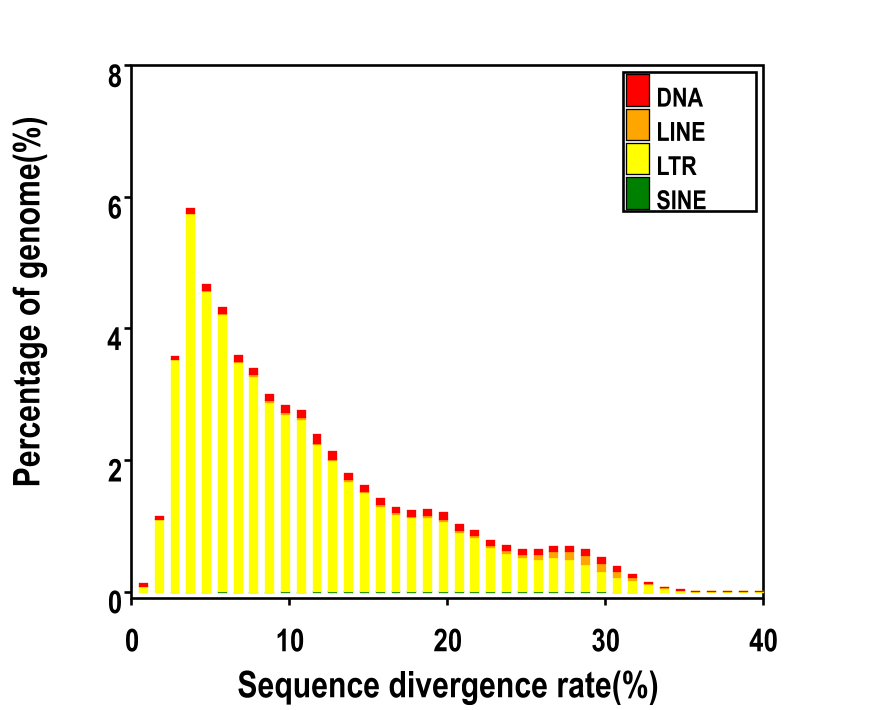
**

Figure S3. Distribution of TE sequences in the genome of *S. brachycarpa*

**
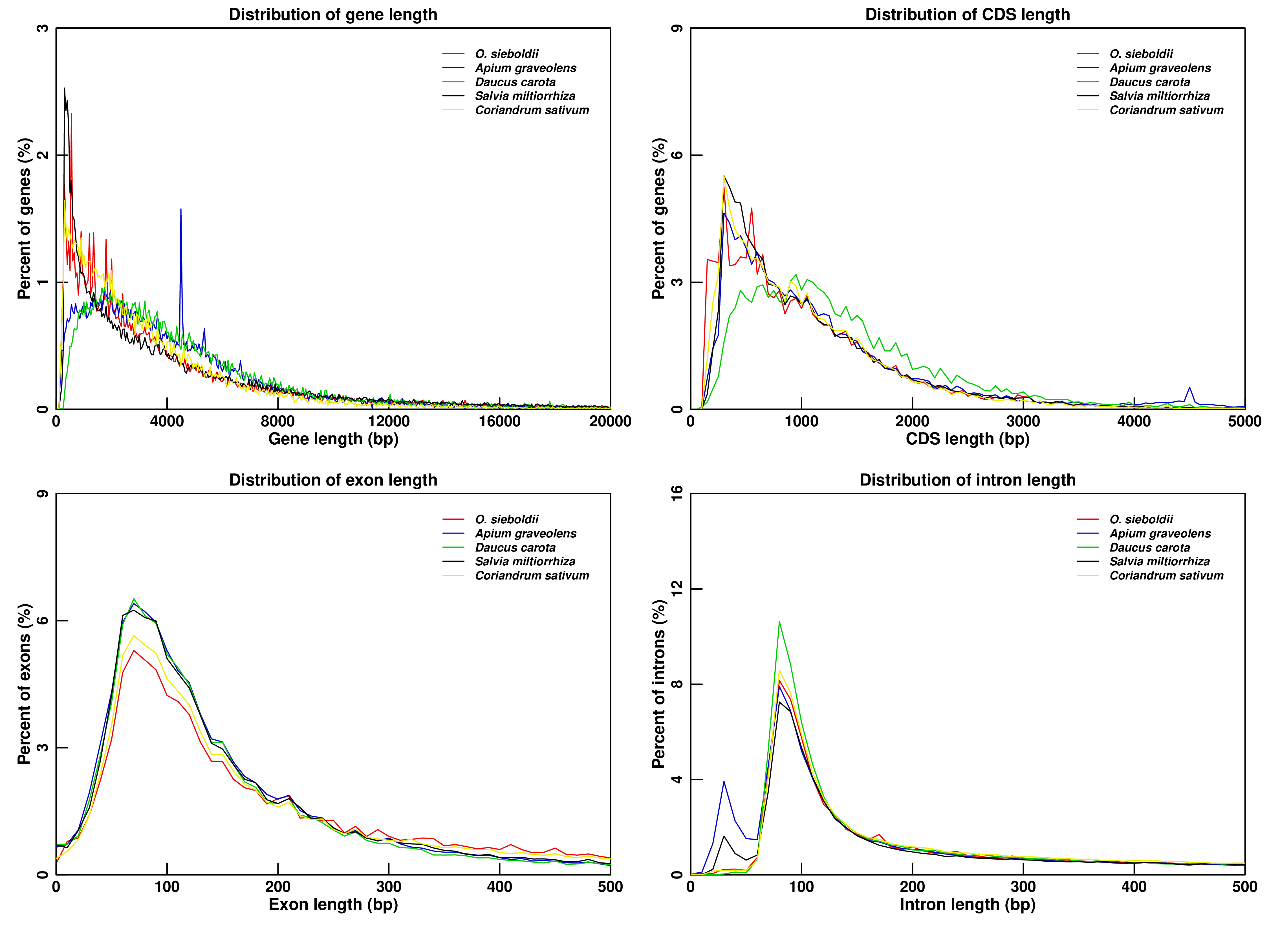
**

Figure S4. Comparison of gene lengths between *S. brachycarpa* and other species.

**
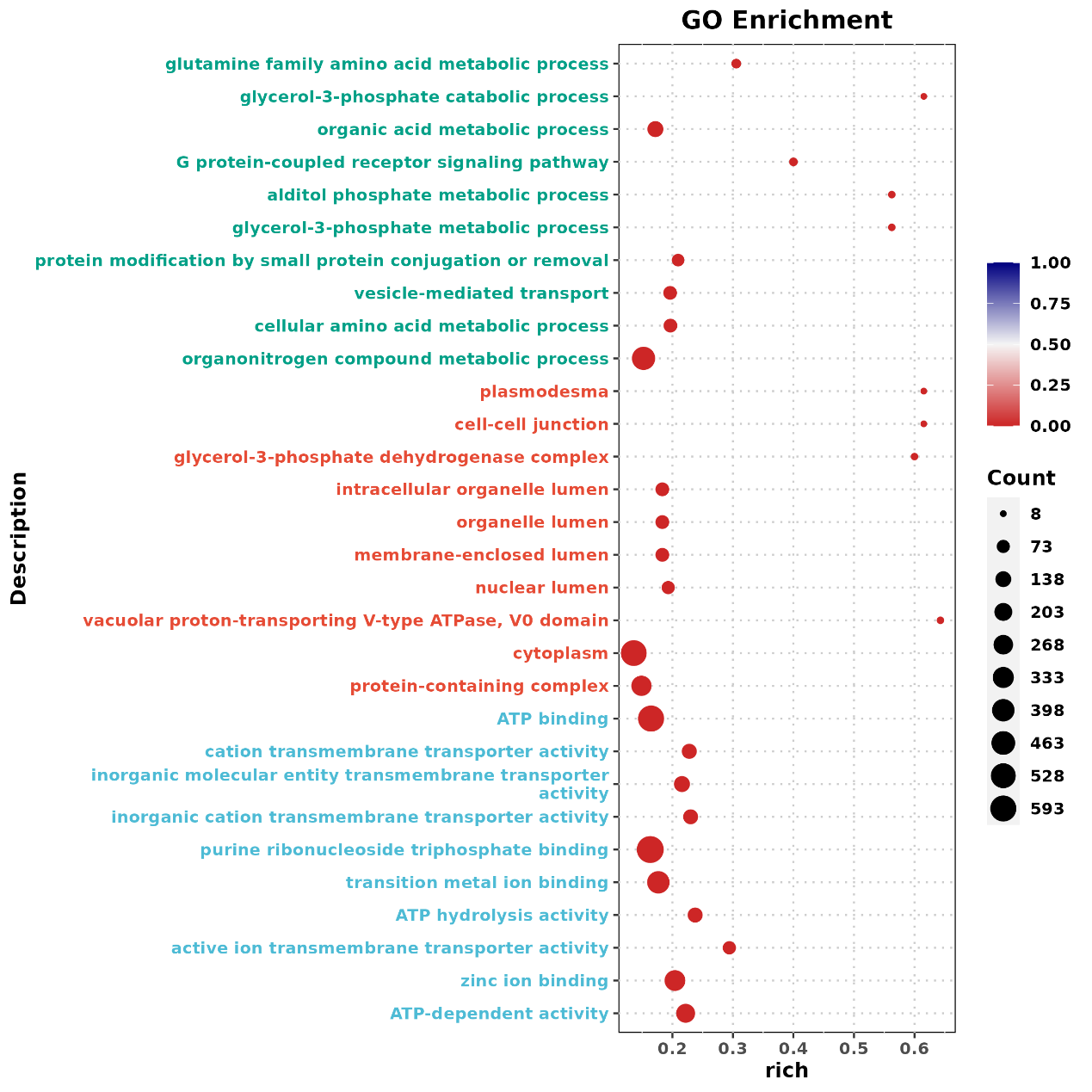
**

Figure S5. GO enrichment results of *S. brachycarpa* unique gene family.

**
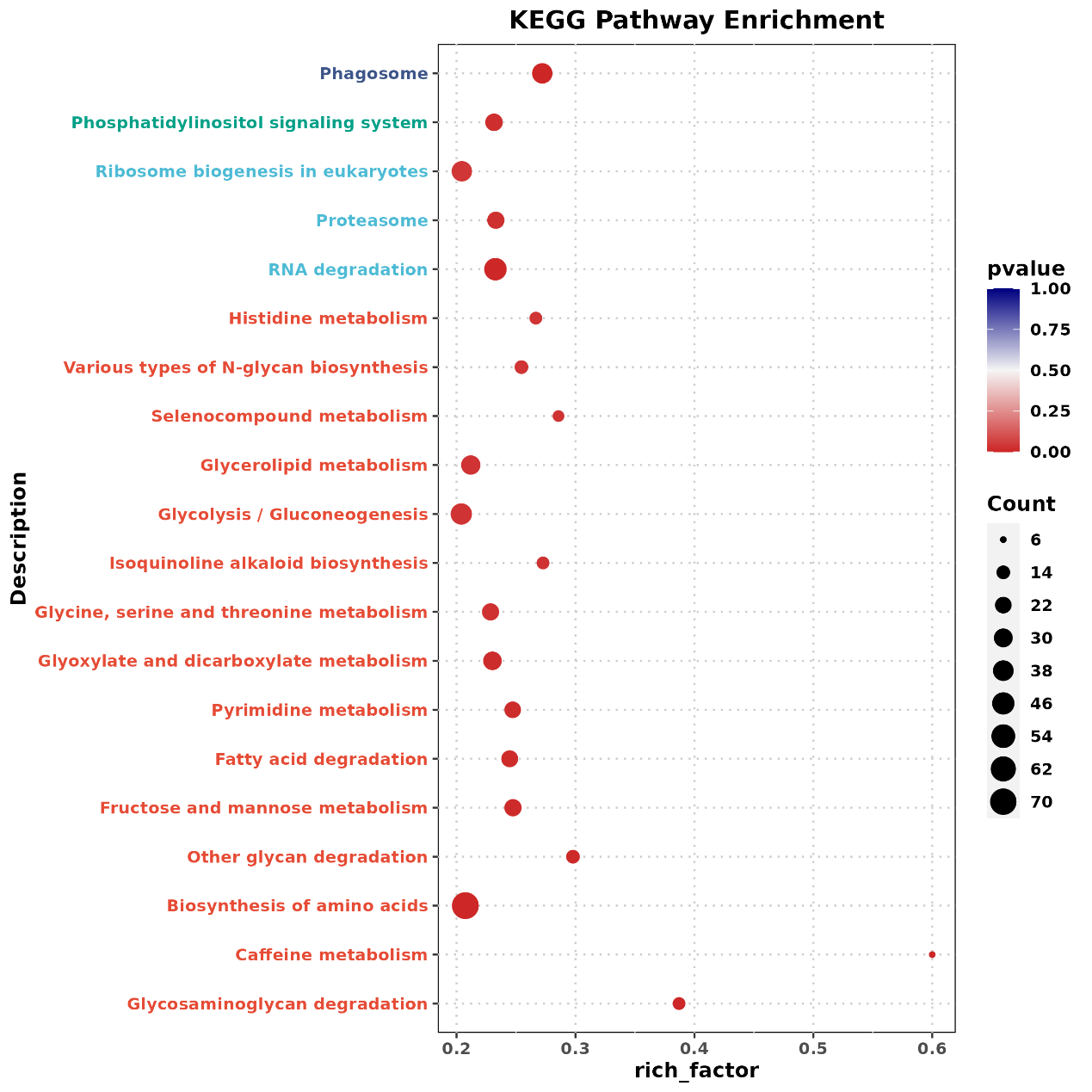
**

Figure S6. KEGG enrichment results of *S. brachycarpa* unique gene family.

**
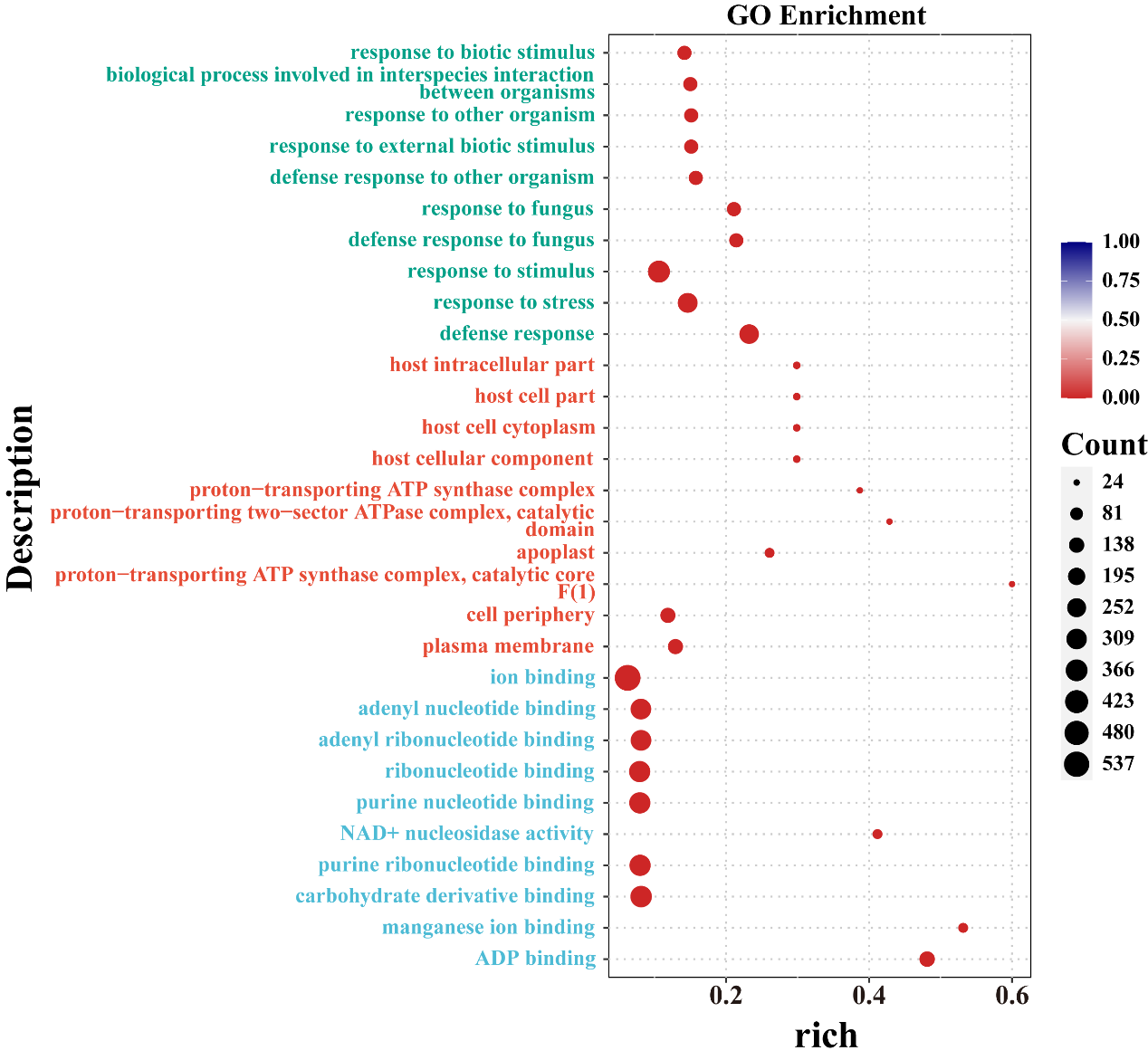
**

Figure S7. GO enrichment results of *S. brachycarpa* expansion gene family.

**
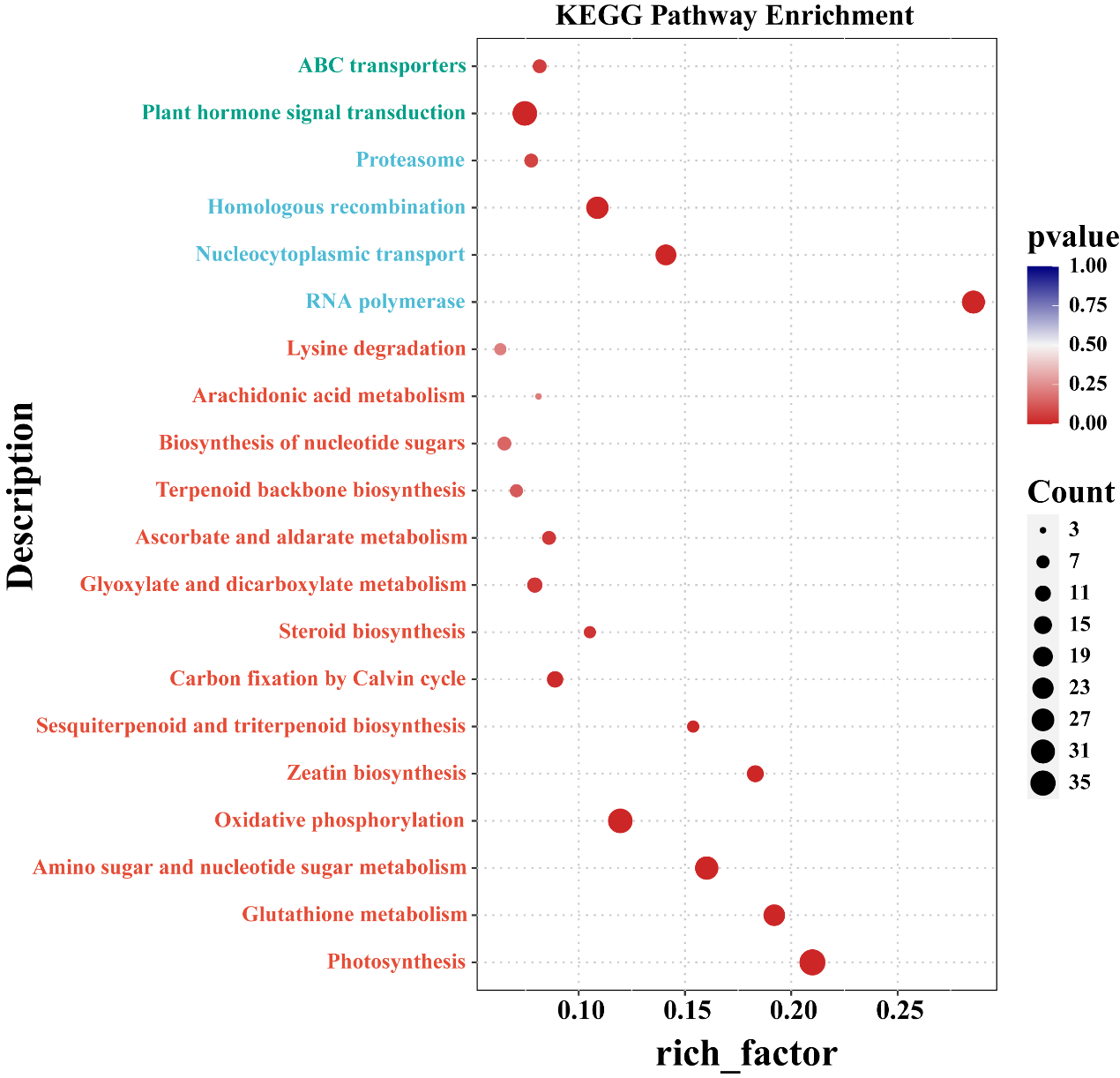
**

Figure S8. KEGG enrichment results of *S. brachycarpa* expansion gene family.

**
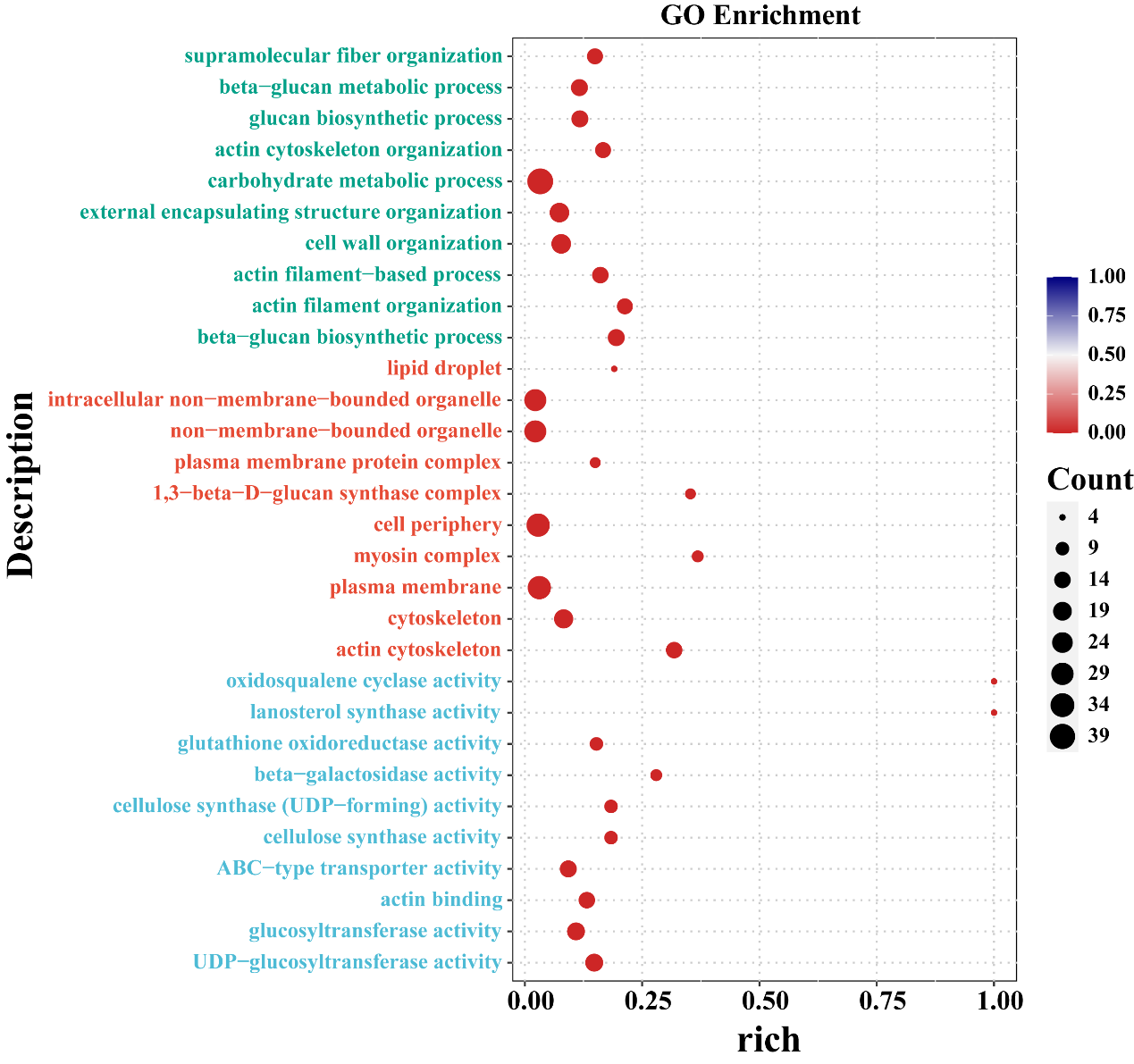
**

Figure S9. GO enrichment results of *S. brachycarpa* contraction gene family.

**
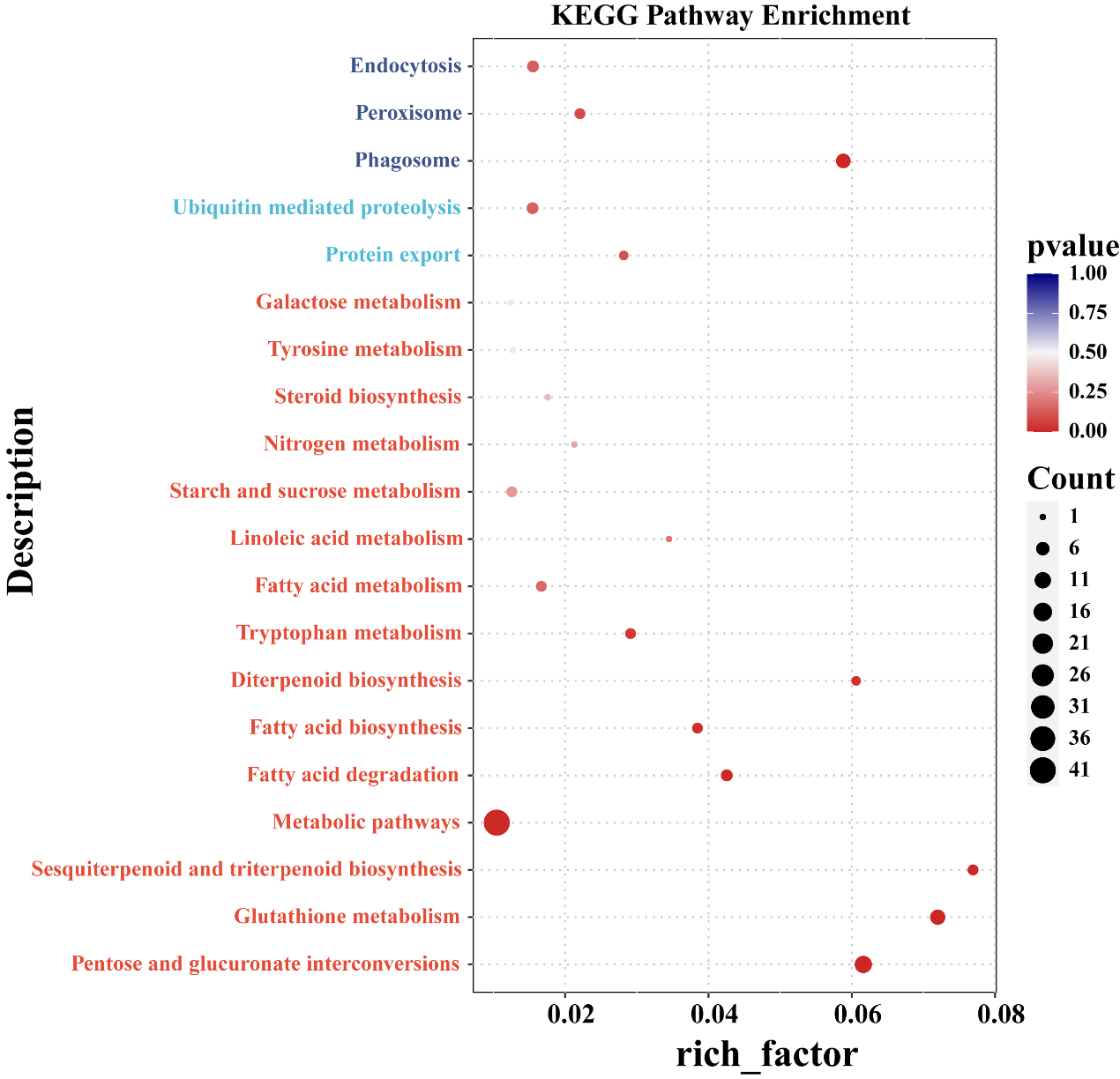
**

Figure S10. KEGG enrichment results of *S. brachycarpa* contraction gene family.

**
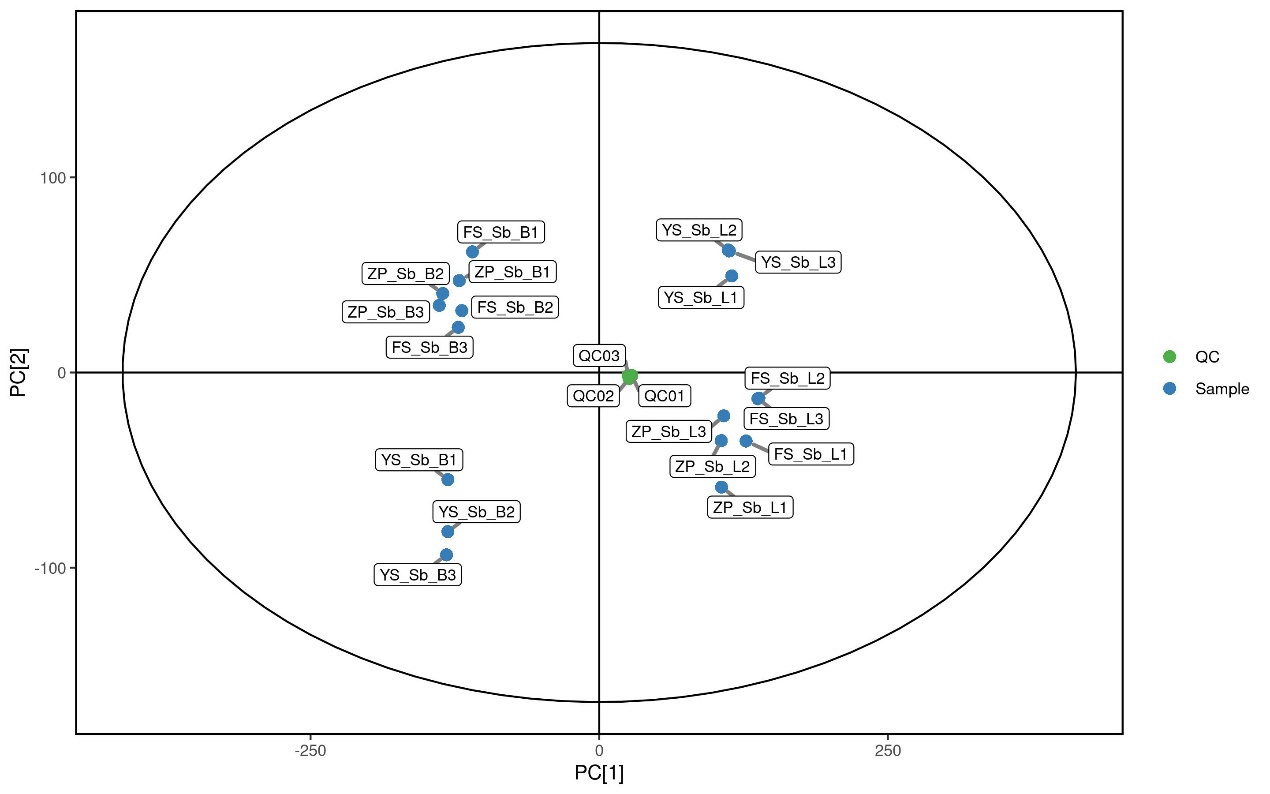
**

Figure S11. PCA diagram of *S. brachycarpa* sample at three different habitats, including Wild Habitat (YS), Cultivated Habitat (ZP), and Understory Bionic Habitat (FS). "L" represents leaf, and "B" represents petiole.


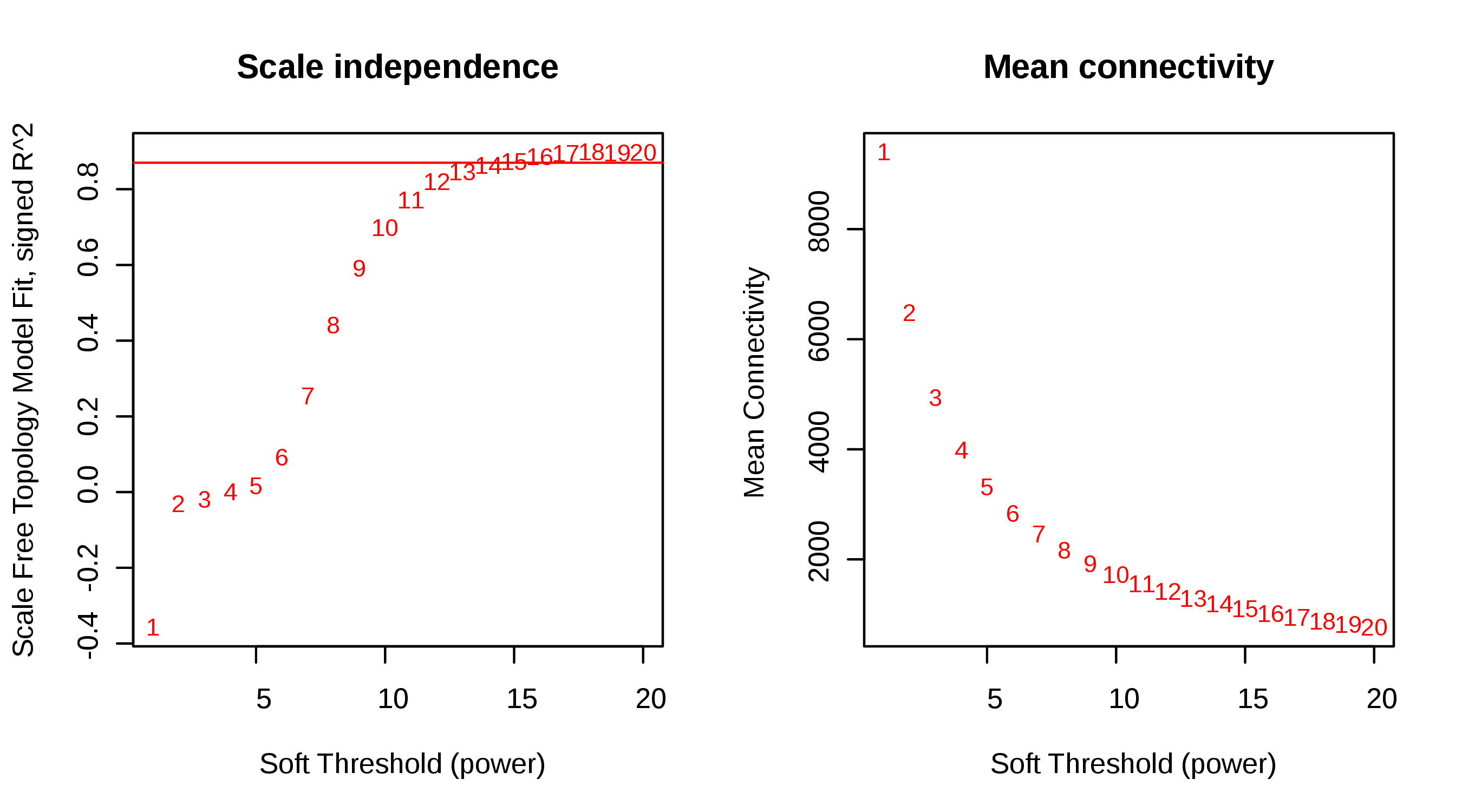


Figure S12: Scale-free topology fit analysis. The plot illustrates the trends of the scale-free topology fit index (R², left panel) and the mean connectivity of the network (right panel) as the soft-thresholding power increases from 1 to 20.


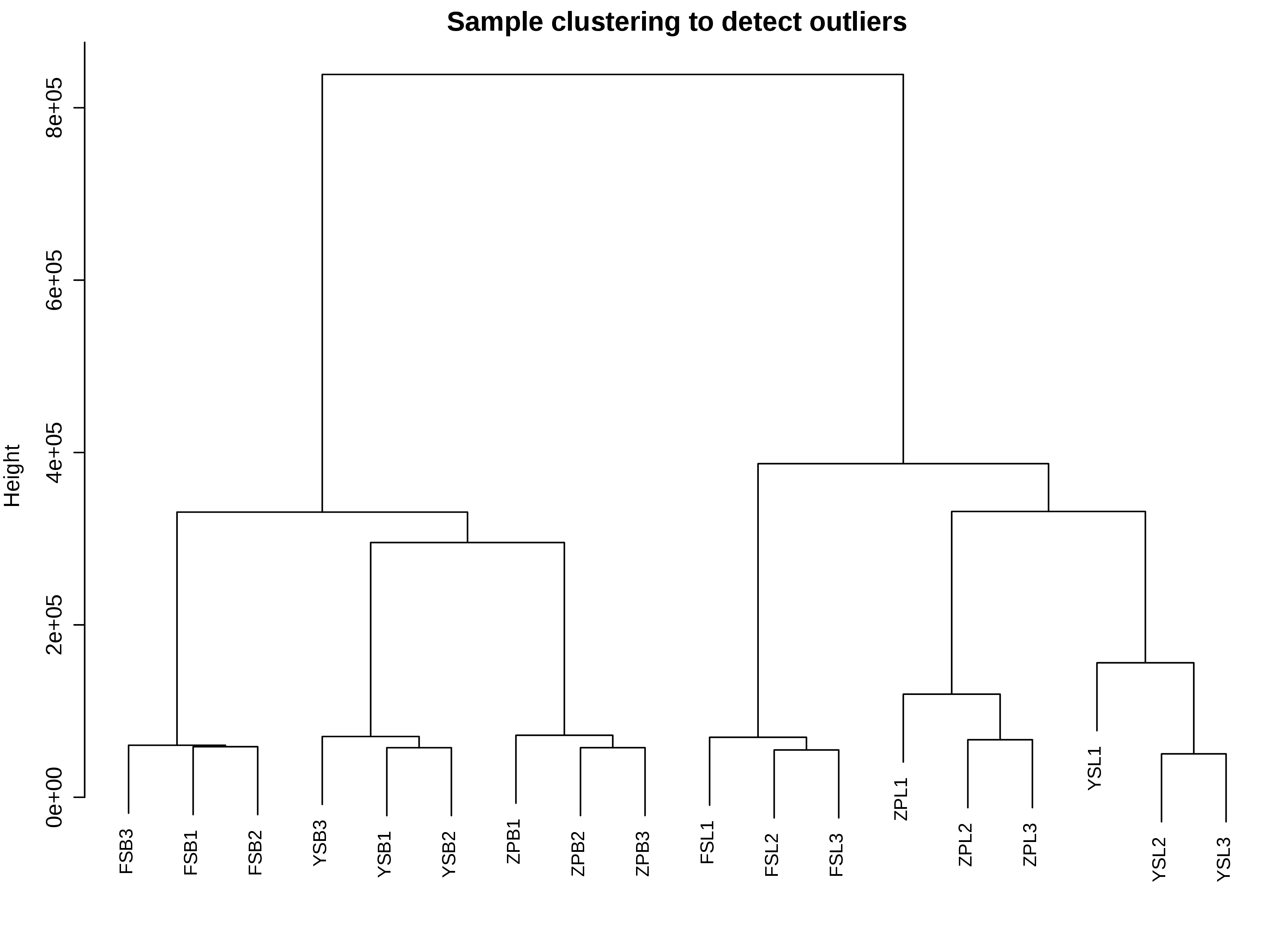


Figure S13: Sample clustering dendrogram. Shown is the hierarchical clustering of samples derived from the global gene expression matrix.


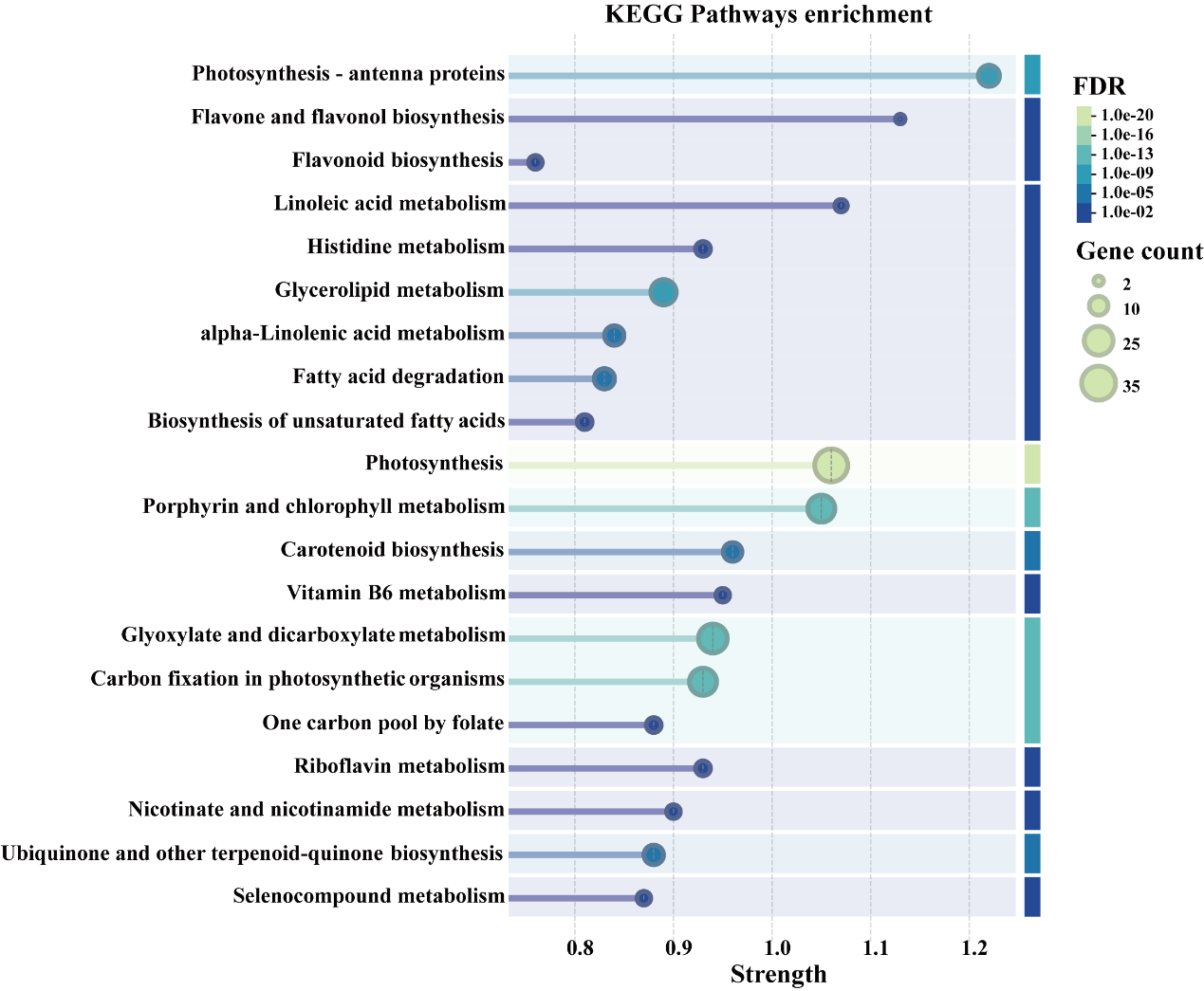


Figure S14. KEGG enrichment analysis of genes in in the blue module**
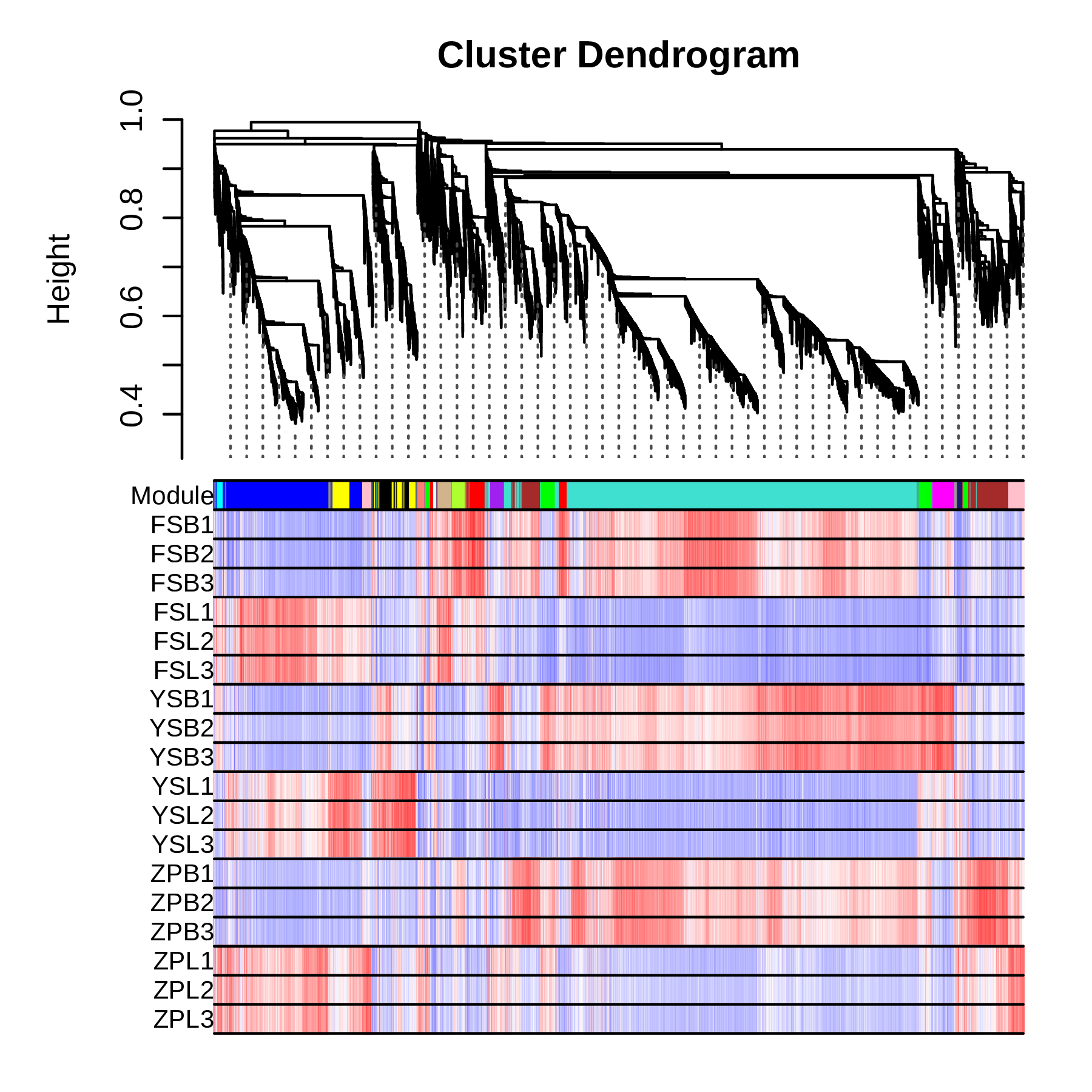
**

Figure S15. Clustering heat map of gene expression in each module.


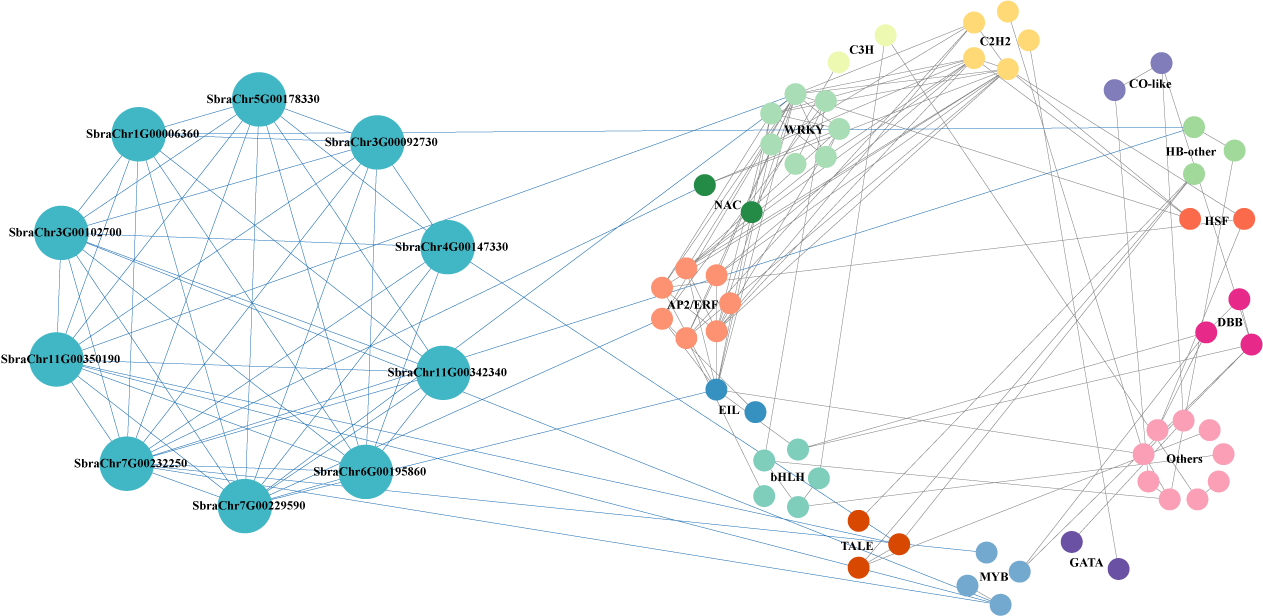


Figure S16. Predicted transcriptional regulatory network between TFs and genes associated with the flavonoid synthesis pathway in blue module. The network is constructed based on correlation analysis and predictions from the String database, representing predicted regulatory relationships.


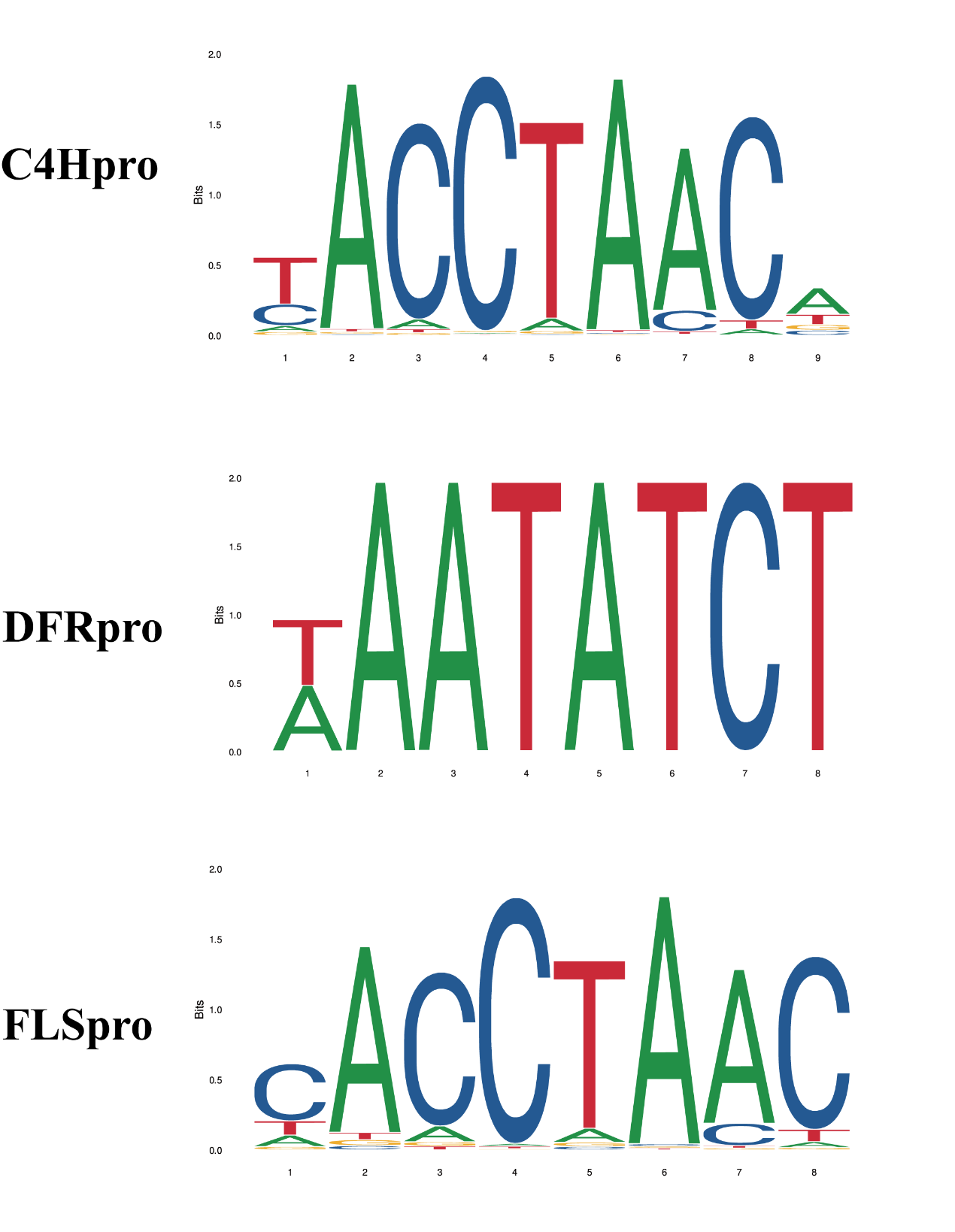


Figure S17. WebLogo of predicted MYB binding motifs in the *C4H*pro, *DFR*pro, and *FLS*pro promoters of *S. brachycarpa.*


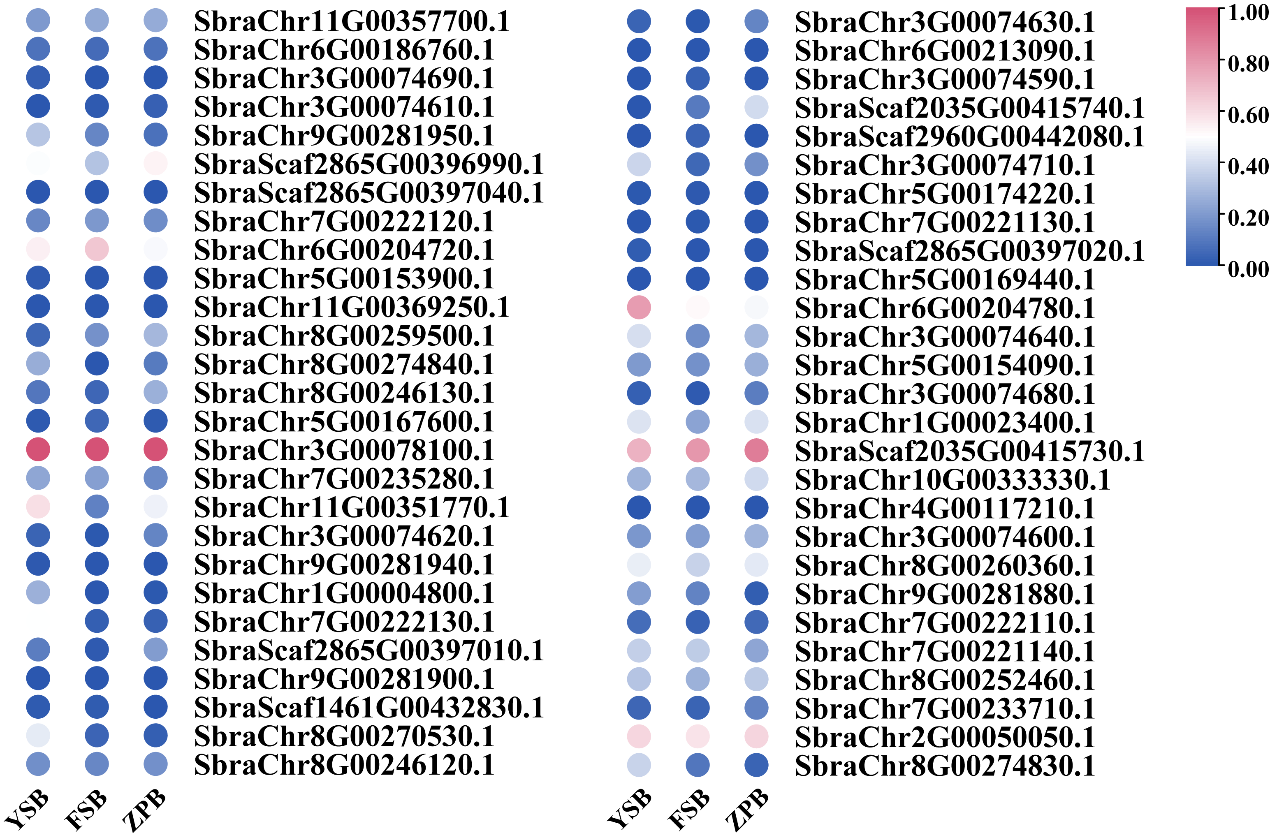


Figure S18. Expression heatmap of the TPS gene family in *S. brachycarpa* petiole tissues under three habitats.


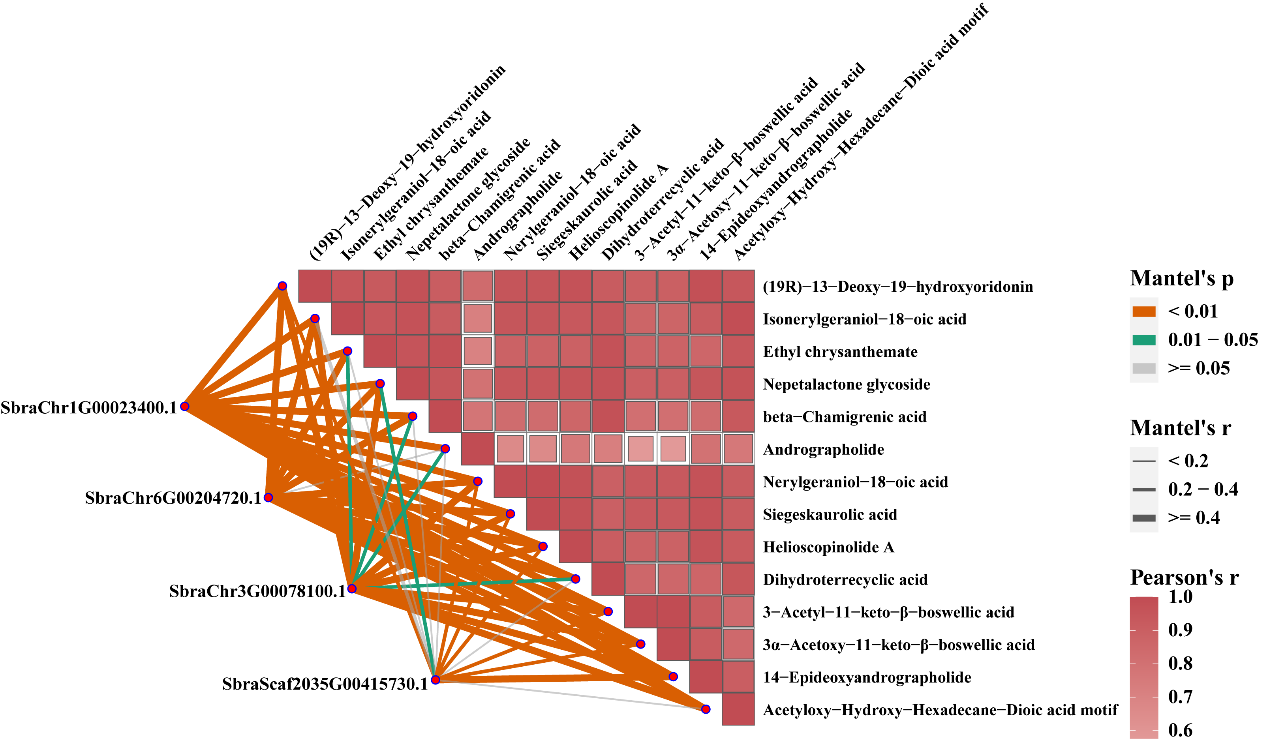


Figure S19. Correlation analysis between co-expressed TPS genes and terpenoids representative components in *S. brachycarpa* under three habitats.


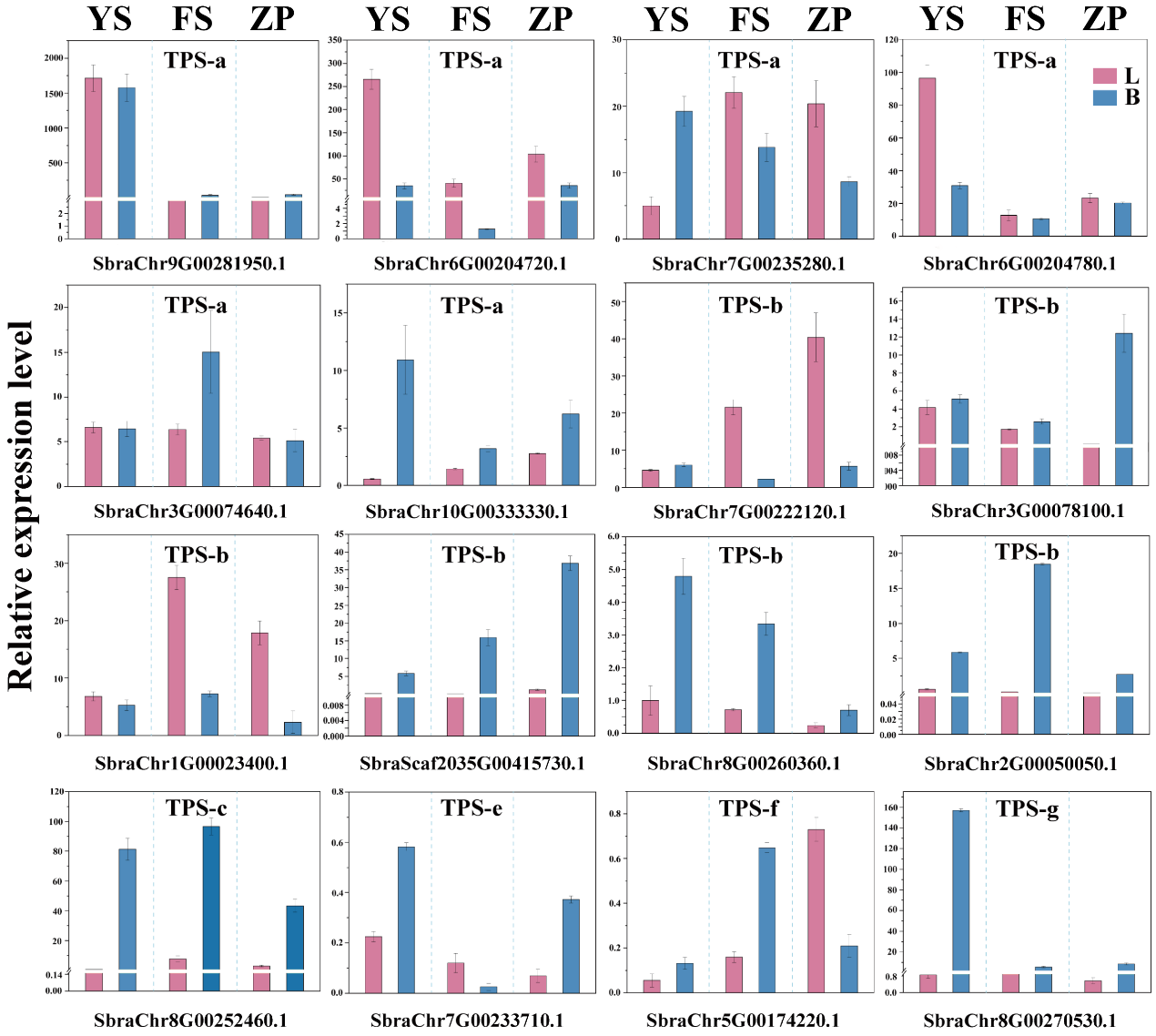


Figure S20. Relative expression levels of *TPS* genes of *S. brachycarpa* sample at three different habitats by RT-qPCR, including Wild Habitat (YS), Cultivated Habitat (ZP), and Understory Bionic Habitat (FS). "L" represents leaf, and "B" represents petiole.
